# The ER-Membrane Transport System Is Critical for Intercellular Trafficking of the NSm Movement Protein and Tomato Spotted Wilt Tospovirus

**DOI:** 10.1371/journal.ppat.1005443

**Published:** 2016-02-10

**Authors:** Zhike Feng, Fan Xue, Min Xu, Xiaojiao Chen, Wenyang Zhao, Maria J. Garcia-Murria, Ismael Mingarro, Yong Liu, Ying Huang, Lei Jiang, Min Zhu, Xiaorong Tao

**Affiliations:** 1 Department of Plant Pathology, Nanjing Agricultural University, Nanjing, People's Republic of China; 2 Departament de Bioquímica i Biologia Molecular, Universitat de València, Burjassot, Spain; 3 Institute of Plant Protection, Hunan Academy of Agricultural Sciences, Changsha, People's Republic of China; Agriculture and Agri-Food Canada, CANADA

## Abstract

Plant viruses move through plasmodesmata to infect new cells. The plant endoplasmic reticulum (ER) is interconnected among cells via the ER desmotubule in the plasmodesma across the cell wall, forming a continuous ER network throughout the entire plant. This ER continuity is unique to plants and has been postulated to serve as a platform for the intercellular trafficking of macromolecules. In the present study, the contribution of the plant ER membrane transport system to the intercellular trafficking of the NSm movement protein and *Tomato spotted wilt tospovirus* (TSWV) is investigated. We showed that TSWV NSm is physically associated with the ER membrane in *Nicotiana benthamiana* plants. An NSm-GFP fusion protein transiently expressed in single leaf cells was trafficked into neighboring cells. Mutations in NSm that impaired its association with the ER or caused its mis-localization to other subcellular sites inhibited cell-to-cell trafficking. Pharmacological disruption of the ER network severely inhibited NSm-GFP trafficking but not GFP diffusion. In the *Arabidopsis thaliana* mutant *rhd3* with an impaired ER network, NSm-GFP trafficking was significantly reduced, whereas GFP diffusion was not affected. We also showed that the ER-to-Golgi secretion pathway and the cytoskeleton transport systems were not involved in the intercellular trafficking of TSWV NSm. Importantly, TSWV cell-to-cell spread was delayed in the ER-defective *rhd3* mutant, and this reduced viral infection was not due to reduced replication. On the basis of robust biochemical, cellular and genetic analysis, we established that the ER membrane transport system serves as an important direct route for intercellular trafficking of NSm and TSWV.

## Author Summary

Plant viruses may use different host cell transport machineries to move from one cell to another through plasmodesmata. The contribution of host cell transport systems to the intercellular movement of multipartite negative-strand RNA plant viruses including tospoviruses is poorly understood. We used *Tomato spotted wilt tospovirus* (TSWV) as a model to understand the mechanism of intercellular movement of tospoviruses. In this study, using *in vitro* and *in vivo* systems for characterizing membrane proteins, we identified that the TSWV NSm movement protein was physically associated with the ER membrane. NSm expressed in a single leaf cell was able to move into neighboring cells along the ER membrane network. The ER membrane in plants is a unique structure that runs between neighboring cells via the ER desmotubule of the plasmodesmata and forms a continuous network throughout the plant. Taking advantage of TSWV NSm being tightly associated with ER membrane and trafficked between cells through plasmodesmata, we demonstrated here by robust biochemical, cellullar and genetic evidence that the ER membrane transport system of plants serves as an important route for intercellular trafficking of the NSm movement protein and TSWV. Our findings have important new implications for mechanistic studies on intercellular trafficking of tospoviruses and other multipartite negative-strand RNA plant viruses.

## Introduction

Plasmodesma-mediated macromolecular trafficking plays important roles in plant growth and development [1–3] and in plant–pathogen interactions [4–6]. Structurally, a plasmodesma is composed of the plasma membrane with a central, modified appressed endoplasmic reticulum (ER), the desmotubule [7]. Besides the long-established cell-to-cell transport of small molecules via plasmodesmata, macromolecules such as proteins and RNAs have been shown in the last two decades to traffic between cells through plasmodesmata (PD). Such macromolecular trafficking is crucial for viral infection [4–6], plant defense [8,9], and developmental regulation [1–3].

Plant viruses need to move within and between cells to establish systemic infection. To accomplish this task, the plant virus encodes a movement protein (MP) to facilitate intracellular trafficking of the viral genomes from the replication site to PD and to assist the spread of the viral replication complexes or viral particles between plant cells through PD [5,6,10–13]. Plant viruses not only utilize viral-encoded MPs or other viral components for viral intra- and intercellular movement, but also co-opt host cell transport machineries for their movement [13–17]. The cytoskeleton and membrane transport systems of cells are important for intracellular movement of vertebrate viruses (reviewed in [16]), essential for organellar trafficking within plant cells [18,19] and involved in the intercellular trafficking of macromolecules [20,21]. In the case of the best-studied plant virus, *Tobacco mosaic virus* (TMV), the ER membrane is important for its association with the viral replication complexes (VRC) and MP granules, whereas microtubules and microfilaments facilitated their movement on the ER (reviewed in [22]). The ER membrane also serves as an important platform for anchoring several other viral MPs, which are required for intracellular movement and viral spread [23–27]. The ER-to-Golgi secretory pathway is further involved in PD targeting and intercellular trafficking of several viruses [28–33]. Microfilaments and different myosin motors also participate in the intra- or intercellular movement of diverse MPs or viruses [28–30,34–40]. In addition, the endocytic pathway has also been shown to influence the movement of three viruses [41–43].

We used *Tomato spotted wilt topspovirus* (TSWV) as a model to study the mechanism of intercellular movement of tospoviruses and multipartite negative-strand RNA plant viruses. TSWV, the type member of *Tospovirus* which is the only genus containing plant-infecting negative-strand RNA viruses in the family *Bunyaviridae* [44–46], causes severe diseases in many economic important crops and is listed as one of the most devastating plant viruses worldwide [47]. Its nonstructural protein NSm is not encoded by any of the animal-infecting members of *Bunyaviridae* and is thought to be the result of evolutionary adaptation of tospoviruses to infect plants [48]. It has the typical characteristics of a plant viral MP, including a short period of expression early in systemic infection, association with nucleocapsid aggregates in the cytoplasm [49], localization to PD [50], presence in the cell wall and membrane fractions of cells [49–51], induction of tubule structures [52,53], increasing the size exclusion limit of PD [50,54], binding of nucleic acids [55], complementation of cell-to-cell and long-distance movement of a movement-deficient TMV [51,53] or *Cucumber mosaic virus* (CMV) vector [56] by heterologous expression of NSm and interactions with two host trafficking proteins [55,57]. Recently, the biochemical properties of TSWV and other tospoviruses NSm related to membrane association have been characterized [51,58]. Although these findings have partially elucidated the requirements and mechanism for TSWV movement, little is known about the involvement of host cell transport systems for intercellular movement of the NSm MP and TSWV.

The plant ER is a unique structure that is interconnected among cells via the ER desmotubules in the plasmodesma across the cell wall, forming a continuous ER network throughout the entire plant [18,59]. In the present study, using *in vitro* and *in vivo* systems to characterize membrane association properties of movement proteins, we revealed that TSWV NSm was physically and tightly associated with the ER membrane and trafficked from cell to cell. Taking advantage of these features of TSWV NSm, we investigated the contribution of the plant ER membrane transport system to the intercellular movement of NSm MP and TSWV. With robust biochemical, cellullar and genetic evidence, we demonstrated that the plant ER membrane transport system serves as an important direct route for intercellular trafficking of NSm and TSWV. Our findings have important new implications for mechanistic studies of the intercellular trafficking of tospoviruses and other multipartite negative-strand RNA plant viruses.

## Results

### TSWV NSm is physically associated with membranes

Because several studies have reported that TSWV NSm is associated with membranes [49,51,58], we used various transmembrane (TM) predicting tools to determine the sequence in TSWV NSm that might enable insertion into or association with membranes. The predicted outcome (S1 Table) varied according to the methods used. MPEx [60] and DAS-Tmfilter [61] predicted two TM regions, whereas TMpred, and TopPred identified only one (S1 Table and S1 Fig). TMHMM and ΔG prediction algorithms failed to predict any membrane-spanning domain but identified two hydrophobic regions that encompassed residues 127–158, 161–189 (TMHMM [62]) and 127–153 and 163–192 (ΔG Prediction [63]) (S1 Table and S1 Fig).

**Fig 1:**
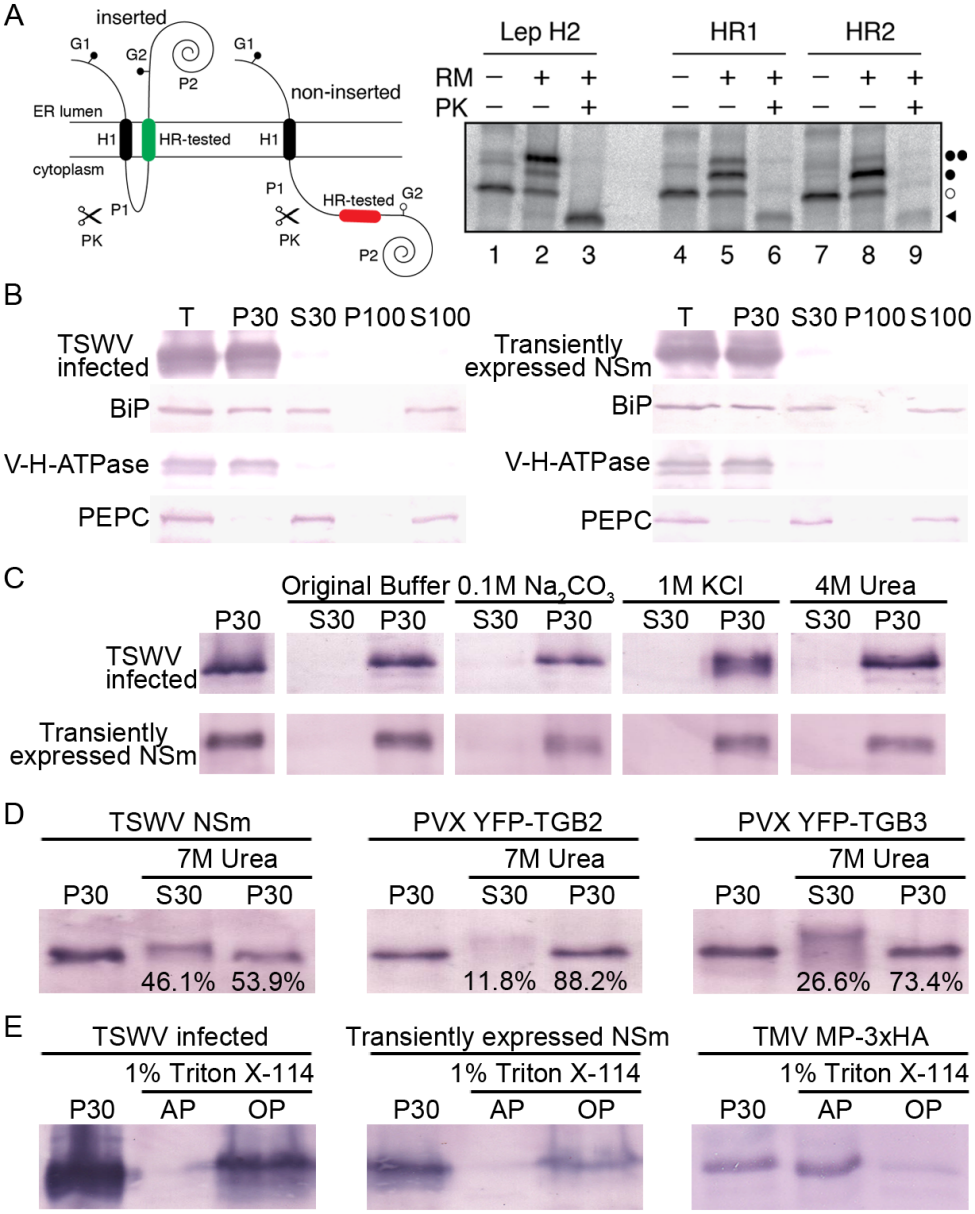
The NSm protein of tomato spotted wilt tospovirus (TSWV) is physically associated with cellular membranes. (A) Insertion assay of NSm hydrophobic regions 1 (HR1) and 2 (HR2) into microsomal membranes using the Lep’ construct. Schematic representation of the Lep-derived construct (Lep’) is shown in upper panel. In this Lep’ construct, H1, derived from the glycosylation acceptor site (G2) at the beginning of the P2 domain, will be modified only if the tested HR inserts into the membrane; the G1 site, embedded in an extended N-terminal sequence of 24 amino acids, is always glycosylated. Results of *in vitro* translation and membrane insertion experiments are shown in the lower panel. Bands of nonglycosylated protein are indicated by a white dot; singly and doubly glycosylated proteins are indicated by one and two black dots, respectively. The protected glycosylated HRs/P2 fragment is indicated by a black triangle. (B) Association of NSm with membrane factions. Total lysate (T) from TSWV-infected or NSm expressing leaves were fractionated into 30,000 × *g* pellet (P30), 30,000×*g* supernatant (S30), 100,000×*g* pellet (P100) and 100,000×*g* supernatant (S100), and analyzed by immunoblots using antibodies against NSm. The vacuolar H-ATPase (V-H-ATPase) subunit E, phosphoenolpyruvate carboxylase (PEPC) and the luminal binding protein (BiP) were used as a microsomal marker, soluble marker and ER marker, respectively, in the fractionation analysis. (C) Biochemical characterization of NSm associated with membranes. The P30 pellet fraction was treated with original lysis buffer, 0.1 M Na_2_CO_3_, 1 M KCl, or 4 M urea, respectively, then separated into supernatant (S30) and pellet (P30) fractions and analyzed by immunoblots using anti-NSm antibodies. (D) Membrane association analysis of TSWV NSm, PVX TGB2 and TGB3 after treatment with 7 M urea. The percentage of proteins eluted in the S30 supernatant or remaining in the P30 pellet, are given at the bottom of the corresponding lanes. (E) Triton X-114 partitioning analysis of TSWV NSm and TMV MP. P30 pellet, aqueous phases (AP) and organic phases (OP) were analyzed by immunoblotting using anti-NSm and anti-HA antibodies, respectively.

To address whether NSm can insert into biological membranes, the two hydrophobic regions identified by the ΔG Prediction was tested using an *in vitro* experimental system that accurately reports the integration of TM helices into microsomal membranes and has been proved to characterize the membrane-spanning capacity of several plant viral MPs [64–67]. This system uses ER-derived microsomal membranes and provides a sensitive way to detect the insertion or translocation of hydrophobic regions through the Sec translocon [68]. The system is based on the cotranslational glycosylation performed by the oligosaccharyltransferase (OST) enzyme. OST adds sugar residues to consensus sequences after the protein emerges from the translocon channel. The glycosylation of a protein region translated *in vitro* in the presence of microsomal membranes therefore indicates the exposure of this region to the OST active site on the luminal side of the ER membrane. In our experimental assay (Fig 1A), a hydrophobic segment to be assayed (HR-tested) replaces the second TM segment from the integral membrane protein Lep. The glycosylation acceptor site (G2) located at the beginning of the P2 domain will be modified only if the HR-tested segment inserts into the membrane, while the G1 site, embedded in an extended N-terminal sequence is always glycosylated [69,70]. We found that HR1 and HR2 of NSm inserted 27.04 ± 4.53% and 14.24 ± 3.99%, respectively, of the molecules into the membrane. The nature of the cytosolic/luminal domains was further examined by proteinase K (PK) digestions. Treatment with PK degrades domains of membrane proteins that protrude into the cytosol, but membrane-embedded or luminally exposed domains are protected. The addition of PK to the Lep-derived translation mixtures (Fig 1A, lanes 6 and 9) generated a residual band originating from the HR2-containing construct, which corresponded to the protected, glycosylated HR1-P2 fragment. These results suggest that NSm hydrophobic regions insert more efficiently than the corresponding regions of TMV and PNRSV MPs [65,66], but not as well as the membrane-spanning capacity required for TM disposition that has been observed for plant viruses with several small integral MPs [64,67].

The microsomal *in vitro* system closely mimics the conditions of *in vivo* membrane protein assembly. However, HR-tested sequences are not analyzed in their native context. To further characterize the membrane-association of full-length TSWV NSm protein, we prepared subcellular fractions (30,000 × *g* pellet [P30], 30,000 × *g* supernatant [S30], 100,000 × *g* pellet [P100] and 100,000 × *g* supernatant [S100]) from *N*. *benthamiana* plants that were infected with TSWV or that transiently expressed the NSm protein. The ER luminal marker (luminal binding protein, BiP) was localized in both the microsomal and soluble fractions (Fig 1B). The vacuolar H-ATPase (V-H-ATPase) subunit E was present primarily in the microsomal P30 fraction, whereas soluble phosphoenolpyruvate carboxylase (PEPC) was found exclusively in the soluble fractions (Fig 1B). Immunoblotting showed that NSm was mainly present in the P30 membrane fraction (Fig 1B). These results agreed with earlier data suggesting that TSWV NSm is a membrane-associated protein [49,51,58].

Alkaline extraction (0.1 M Na_2_CO_3_), salt (1 M KCl) and 4 M urea treatments have been used to distinguish between peripheral and integral membrane proteins [65,71]. After these chemical treatments, the TSWV NSm MP was recovered from the P30 fractions (Fig 1C) upon centrifugation of both TSWV-infected or transiently expressing NSm plant extracts, indicating that NSm protein is tightly associated with cellular membranes. Next, we washed the membranes with 7 M urea, a treatment that should release all polypeptides from the membrane, except for truly integral membrane proteins [65]. As shown in Fig 1D, 46.1% of NSm protein was detected in the soluble fraction. In control experiments, 11.8% of the small integral MPs TGB2 and 26.6% of the TGB3 of *Potato virus X* (PVX) [27,72,73] were detected. These results suggested that the amount of protein that was released from the membrane fraction for NSm by 7 M urea was lower than for peripheral membrane proteins [65], but more than for truly integral membrane proteins (Fig 1D).

To further investigate whether NSm is physically associated with membranes, we analyzed the membrane association capacity of NSm using a Triton X-114 partitioning assay, in which aqueous and organic phases are formed, and integral membrane proteins should be partitioned into the organic phase [74]. We treated the pellet fraction (P30) from TSWV-infected or NSm-transient-expressing plant materials with the non-ionic detergent Triton X-114 and as the control used TMV MP, which was recently shown to be a peripheral membrane protein [66]. After the Triton X-114 treatment, most of the TMV MP separated into the aqueous phase, as expected for a peripheral protein, whereas NSm from both types of plant samples remained preferentially in the organic phase (Fig 1E). Thus, Triton X-114 partition results clearly showed that NSm is physically associated with membranes.

Considering all the membrane-association data together, we conclude that TSWV NSm is neither a peripheral membrane protein nor a canonical integral membrane protein; instead, the protein is strongly and physically bound to cellular membranes, but its hydrophobic regions probably do not span the lipid bilayer.

### NSm is localized to the ER and PD

**Fig 2:**
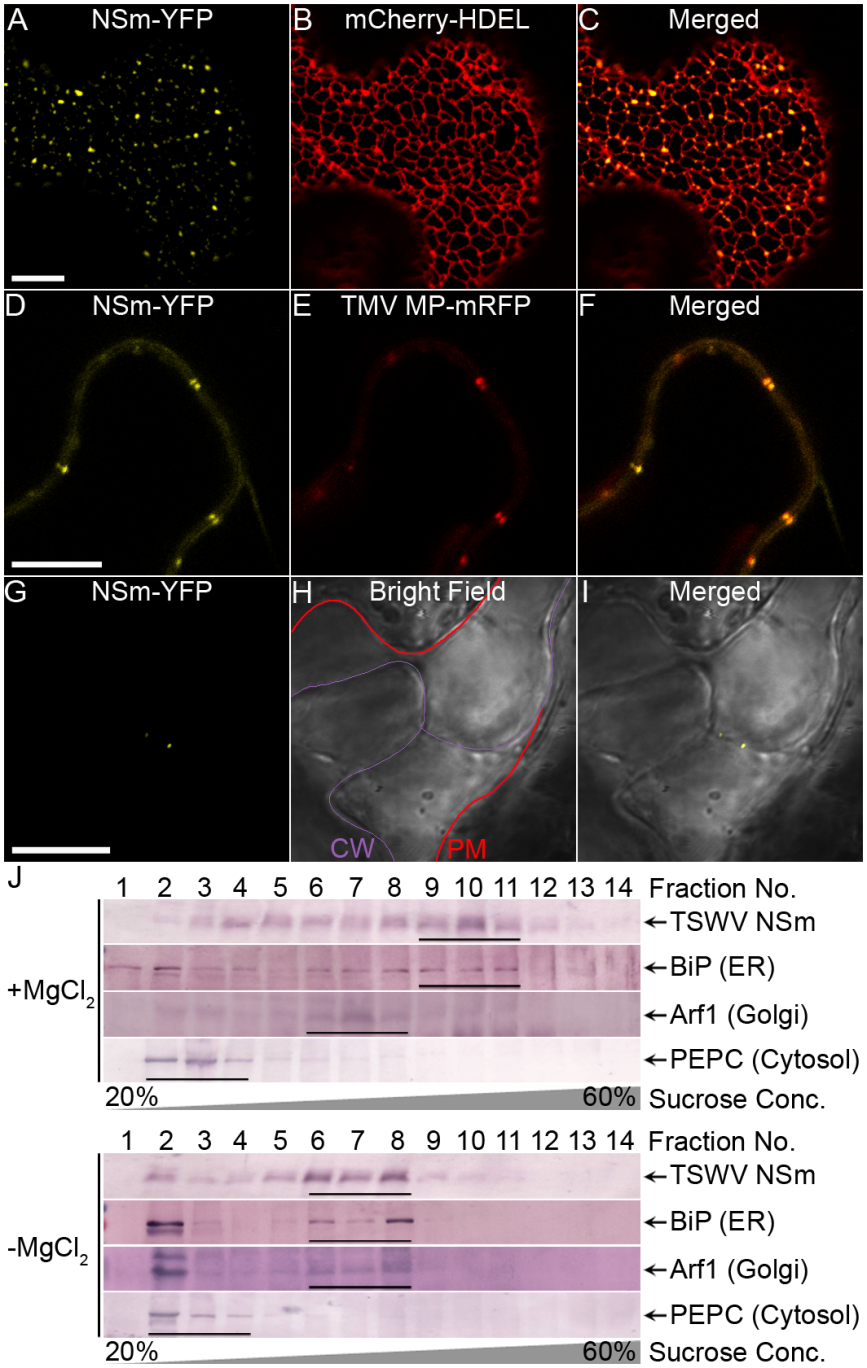
TSWV NSm is localized with the ER and plasmodesmata (PD). (A-C) Colocalization of NSm-YFP with the ER labeled by mCherry-HDEL at 28 h post infiltration (hpi). Bar, 10 μm. (D-F) Colocalization of NSm-YFP with PD labeled by TMV MP-mRFP at 28 hpi. Bar, 10 μm. Different single planes were used to focus on the ER membrane in panels A-C panels and on the periphery of the cell in panels D-F. (G-I) Plasmolysis assay for PD localization of NSm. *N*. *benthamiana* leaves were agroinfiltrated with NSm-YFP, then infiltrated with 10% NaCl at 28 h post agroinfiltration; plasmolyzed cells in the leaf were immediately examined using CLMS. The cell wall (CW) and cytoplasmic membrane (PM) after plasmolysis are marked, respectively, by a purple line and red line. (J) Cofractionation of NSm protein with ER. Extracts of plants transiently expressing NSm were centrifuged on a 20–60% sucrose gradient in the presence or absence of MgCl_2_. Fractions from top to bottom (1 to 14) were analyzed by immunoblots using anti-NSm, anti-BiP, anti-Arf1 and anti-PEPC antibodies to detect NSm, ER luminal protein, Golgi and soluble protein, respectively.

Previous studies have shown that NSm is localized in the cytoplasm and PD [50,58]. To identify the specific membranous structures in which NSm is localized in living cells, we transiently expressed recombinant NSm-YFP (yellow fluorescent protein) in leaf epidermal cells of *N*. *benthamiana* by agroinfiltration. Using confocal laser scanning microscopy (CLSM), we detected NSm-YFP in a structure that was reminiscent of the ER network (Fig 2A). We also detected NSm-YFP near the cell walls in a punctate pattern reminiscent of PD localization (Fig 2D). Co-expression of NSm-YFP with the cortical ER marker mCherry-HDEL confirmed NSm localization to the ER (Fig 2A–2C). We also checked the localization of NSm-YFP for Golgi bodies (using marker Man49-mCherry), nucleus (H2B-mRFP) and chloroplasts after agroinfiltration of *N*. *benthamiana* leaves. CLMS showed that NSm-YFP did not co-localize with Golgi bodies (S2A–S2C Fig), nucleus (S2D–S2F Fig) or chloroplasts (S2G–S2I Fig).

To verify ER localization of NSm independently, we used sucrose gradient fractionation to analyze membrane association patterns of NSm with materials derived from transiently expressed NSm in *N*. *benthamiana*. To verify the association of NSm with the ER, sucrose gradients fractions with or without MgCl_2_ were prepared from *N*. *benthamiana* leaves transiently expressing NSm. The ER marker BiP, Golgi marker Arf1 and cytoplasmic soluble protein marker PEPC in the fractions were detected by Western blot. In the absence of MgCl_2_, ribosomes become dissociated from the ER, causing a shift in the migration of the ER in the gradient [75]. If the protein is associated with the ER, it will have the same shift as the ER marker BiP in the gradient. Immunoblots of the gradient fractions in the presence or absence of MgCl_2_ revealed that TSWV NSm protein shifted the same as the ER marker BiP; the Golgi marker (Arf1) and the cytosol protein (PEPC) did not shift as the ER did (Fig 2J), These results thus provide additional evidence for ER localization of NSm.

To verify whether the subcellular localization of NSm-YFP in the cell walls represents localization to PD, NSm-YFP and TMV MP, which has been well established to have a PD localization function [76], with red fluorescent protein (RFP) fused at its C-terminus, were co-expressed in *N*. *benthamiana* leaf epidermal cells. The merged images of NSm-YFP and TMV MP-mRFP localization patterns demonstrated that NSm-YFP localized with the PD as did the TMV MP-mRFP marker (Fig 2D–2F). To further validate the PD localization of NSm, we plasmolyzed the cells to separate the cytoplasmic ER from the PD membranes. As shown in Fig 2G–2I, NSm remained on the PD after the cytoplasmic ER was separated from the cell wall by plasmolysis.

Collectively, these results established that NSm is physically associated with ER membranes and recruited to the PD. Thus, we next tested for its potential role in cell-to-cell trafficking.

### Cell-to-cell trafficking of NSm protein

**Fig 3:**
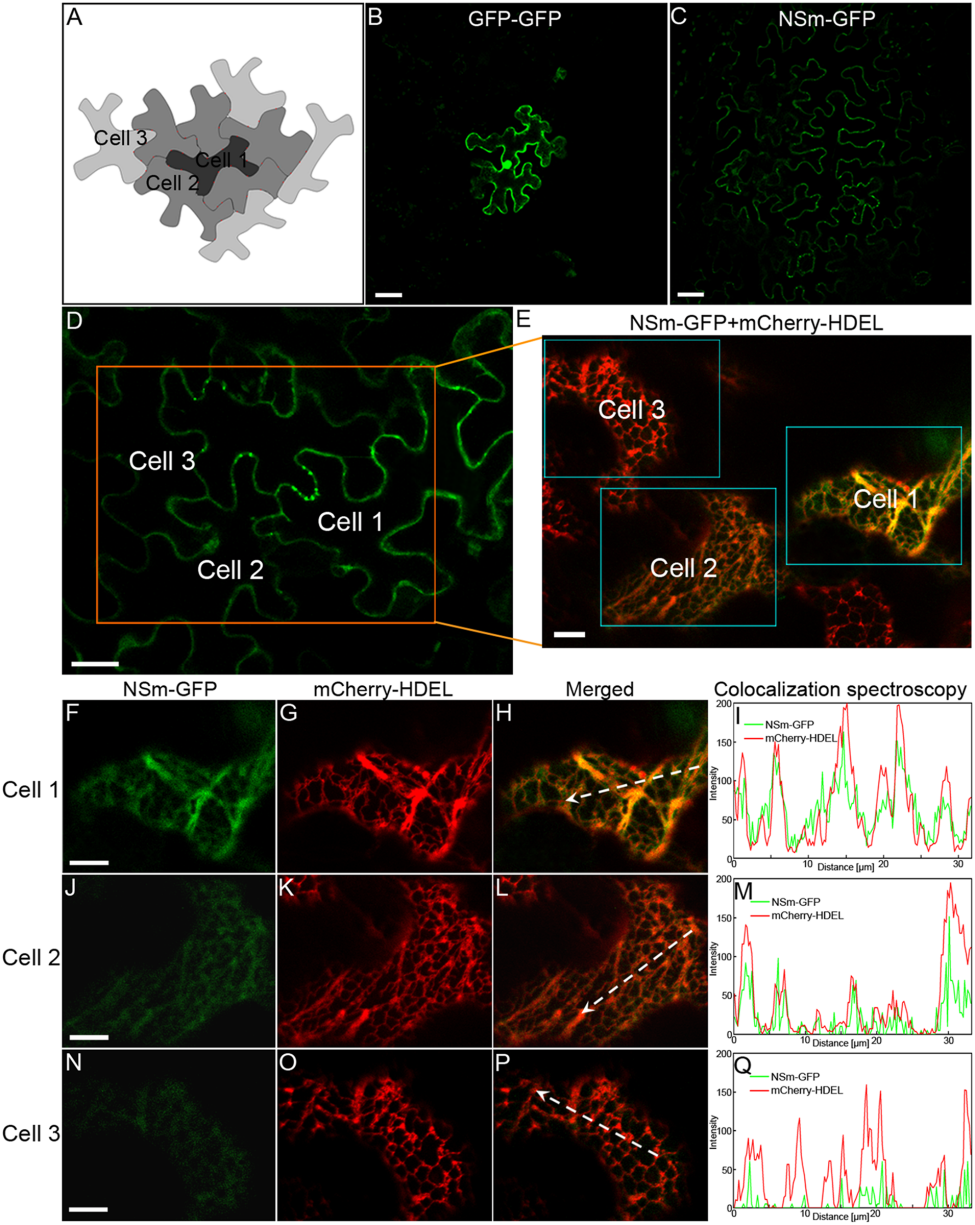
Localization of NSm-GFP in the ER membrane network in bombarded cells with the fusion protein and in neighboring cells receiving the fusion protein in leaf epidermis of *N*. *benthamiana*. (A) Scheme of cell-to-cell transport of NSm in leaf epidermis of *N*. *benthamiana* by bombardment. (B and C) Cell-to-cell movement of GFP-GFP (B) and NSm-GFP (C) in leaf epidermis of *N*. *benthamiana*. Bar, 50 μm. (D-Q) NSm-GFP was localized in the interconnected ER network in cells bombarded with the fusion protein and in neighboring cells that subsequently received the protein. A low magnification image to show that NSm moved intercellularly after bombardment (D). Bar, 20 μm. A region with three cells showing NSm movement (Cell 1 to Cell 3) in image D was magnified (boxed region) to show colocalization of NSm-GFP with the ER labeled by mCherry-HDEL (E). Bar, 10 μm. The boxed region in image E corresponding to the respective initially bombarded cell (Cell 1) and the second (Cell 2) and third layer (Cell 3) of cells into which NSm moved was further split into three channels to show colocalization of NSm-GFP with the ER labeled by mCherry-HDEL (Cell 1, F-H; Cell 2, J-L; Cell 3, N-P). Colocalization of NSm and ER in the respective cells was further analyzed by overlapping fluorescence spectra (I, M and Q). Bar, 10 μm.

To test whether NSm functions in cell-to-cell trafficking, we bombarded epidermal cells on intact leaves of *N*. *benthamiana* plants with a DNA construct that expresses an NSm-GFP fusion protein. As a control, we bombarded separate leaves with a DNA construct expressing a GFP-GFP fusion protein. As shown in Fig 3B and Table 1, GFP-GFP (54.0 kDa) was restricted to single cells in a total of 53 fluorescent loci expressing the fusion protein at 22 h post-bombardment. At the same time, NSm-GFP (60.9 kDa) was trafficked into neighboring cells in 58 of the 110 fluorescent foci expressing the fusion protein (Fig 3C and Table 1). We also analyzed cell-to-cell movement of NSm-GFP and GFP-GFP over 0 h to 48 h post bombardment in epidermal cells of *N*. *benthamiana*. As shown in S2 Table, starting at 9–10 h post bombardment, cell-to-cell movement of NSm-GFP was detected with 16.3% of the foci expressing the fusion protein. From 10 to 21 h post bombardment, NSm-GFP continued to move from the initial site, reaching 34.5% of the foci at 14–15 h, 40.4% at 19–20 h and 41.9% at 26–27 h. At 48 h post bombardment, the cell-to-cell movement of NSm-GFP had decreased to 27.4% of the foci (S2 Table). Parallel observations showed that GFP-GFP remained in the initial cell. To test whether the addition of NSm can facilitate the cytoplasmic soluble GFP-GFP protein to traffick into neighboring cells, leaves of *N*. *benthamiana* were agroinfiltrated to express NSm or an empty vector, and 12 h later, bombarded the agroinfiltrated leaves with GFP-GFP. After an additional 24 h, we checked bombarded foci for cell-to-cell movement of GFP-GFP. As shown in S3 Table, GFP-GFP did not move from the initially bombarded cell in the presence of NSm.

**Table 1:** Cell-to-cell movement of TSWV NSm in leaf epidermis of *Nicotiana benthamiana* after bombardment.

Bombarded plasmid	Protein size (kDa)	Total no. of signal clusters	No. of clusters with 1 cell (% of total)	No. of clusters with ≥2 cells (% of total)	*P*-value ^b^
**GFP-GFP**	54.0	53	53 (100**%**) ^a^	0	
**NSm-GFP**	60.9	110	52 (47.3**%**)	58 (52.7**%**)	< 0.001
**NSm** ^**133-135D**^ **-GFP**	60.9	84	84 (100**%**)	0	
**NSm** ^**177-179D**^ **-GFP**	60.9	92	92 (100**%**)	0	
**NSm** ^**4A/5A**^ **-GFP**	60.9	106	106 (100**%**)	0	
**NSm** ^**230A/232A**^ **-GFP**	60.9	98	98 (100**%**)	0	

^a^ Signal clusters comprise fluorescent cells, indicating presence of GFP fusion protein.

^b^ *P*-values were calculated using unpaired two-tailed Student *t*-test.

To further verify the cell-to-cell movement of NSm, a plant binary vector was constructed to harbor two gene cassettes to express NSm-GFP and mCherry-HDEL simultaneously (S3A Fig). The agrobacterium containing the plant binary construct was infiltrated at OD_260_ = 0.004 to express it in a single epidermal cell of *N*. *benthamiana*. As shown in S3B Fig (upper panel), the red fluorescence signals from mCherry-HDEL were mostly observed in one to two cells (primary expression cell). Green fluorescence signals from NSm-GFP overlapped with red signals in the primary expression cell, whereas green signals were also observed in cells surrounding the primary cell (secondary expression cells). As a control, we also made a plant binary construct to co-express GFP-GFP and mCherry-HDEL (S3A Fig). When agrobacterium containing this construct was delivered into plant cells using the same strategy for delivering NSm-GFP, both the green fluorescence signals from GFP-GFP and the red fluorescence signals from mCherry-HDEL remained in the primary expression cell; no GFP-only cells were found surrounding the primary expression cell (lower panel in S3B Fig). Thus, these data provide strong evidence that NSm-GFP indeed moves from cell to cell.

As noted already, the plant ER is interconnected among the cells via the desmotubule of the PD, forming a continuous ER network throughout the plant [18,59]. Because NSm is physically associated with the ER, it may move from cell to cell through the PD along the continuous ER network among cells. If so, the trafficked protein is expected to reside in the ER in neighboring cells. To test for this case, we expressed mCherry-HDEL in epidermal leaf cells in *N*. *benthamiana* by agroinfiltration. After 12 h post agroinfiltration, we bombarded *N*. *benthamiana* leaf cells that labeled the ER network with the DNA construct expressing NSm-GFP. After additional 22 h, we checked the cell-to-cell movement of NSm-GFP along the ER membrane. We analyzed a leaf region containing three layers of cells: the initially bombarded cells showing strong fluorescence (Cell 1), the immediately adjacent cells with weaker fluorescence (Cell 2), and the third layer of cells emitting weakest fluorescence (Cell 3) (Fig 3A, 3D and S4A Fig). Merging of the GFP and mCherry channels and a fluorescence spectra analysis showed that NSm-GFP co-localized with mCherry-HDEL in all cells (Fig 3E–3Q and S4 Fig). This pattern is consistent with the hypothesis that NSm-GFP trafficks along the ER network between cells. The following experiments tested this hypothesis directly.

### Membrane integration of NSm is required for its intercellular movement

**Fig 4:**
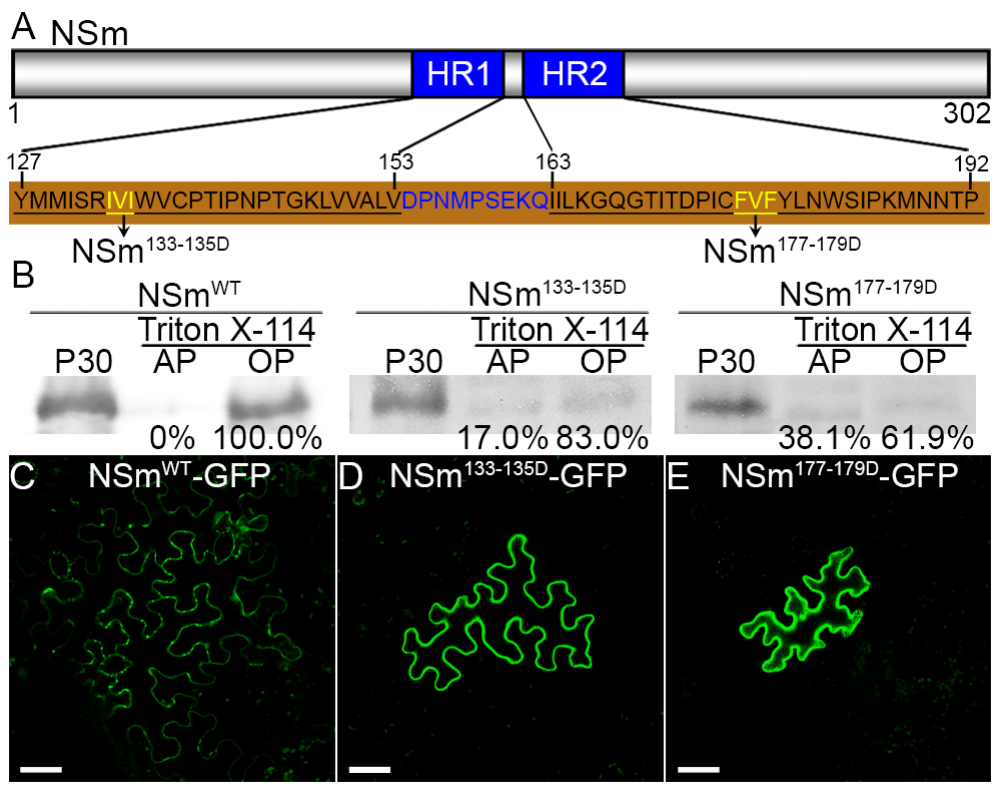
Impairment of membrane integration of NSm inhibits its cell-to-cell movement in leaf epidermis of *N*. *benthamiana*. (A) Diagram of amino acid residues for hydrophobic regions (HR1 to HR2) and two aspartate substitution mutants at sites of amino acids 133–135 and 177–179 in NSm. (B) Triton X-114 partitioning analysis of NSm^133-135D^ and NSm^177-179D^ aspartate substitution mutants. P30 pellet, aqueous phase (AP) and organic phase (OP) were analyzed by immunoblots using anti-NSm antibodies, respectively. (C-E) Cell-to-cell movement analysis of NSm^133-135D^ (D) and NSm^177-179D^ (E) mutant in *N*. *benthamiana* after bombardment. NSm^WT^ (C) was used as a positive control. Bar, 50 μm.

If NSm trafficked along the ER membrane network from cell to cell, disruption of its physical association with ER should inhibit trafficking. We first tested whether mutating the predicted hydrophobic region (residues 127 to 192, which includes HR1 to HR2) of NSm (Fig 4A) would affect its physical association with the membrane. We designed two mutants with a respective aspartate substitution at amino acids 133–135 (IVI) or 177–179 (FVF), roughly in the middle of HR1 to HR2 (Fig 4A). *N*. *benthamiana* leaves were agroinfiltrated with these two mutants, then the membrane fractions prepared from these leaves were treated with Triton X-114. As shown in Fig 4B, after the Triton X-114 treatment, approximately 17% of NSm^133-135D^ and 38% of NSm^177-179D^ mutant proteins partitioned into the aqueous phase, whereas 100% of the wild-type NSm remained in the organic phase, suggesting that the physical membrane integration capacity of the two mutants was altered to a certain degree.

To test the cell-to-cell trafficking function of these two mutants, we examined single cells of *N*. *benthamiana* leaves bombarded with one or the other mutant. Both mutants remained in single cells (Fig 4C–4E and Table 1). Thus, full membrane integration is critical for NSm to traffic from cell to cell, providing another piece of critical experimental evidence to support the hypothesis that NSm traffics intercellularly along the ER network. The following experiments further tested this hypothesis using complementary approaches.

### Mutations in NSm that block its sorting to the cortical ER or cause its mis-sorting to other subcellular localizations inhibit cell-to-cell trafficking

**Fig 5:**
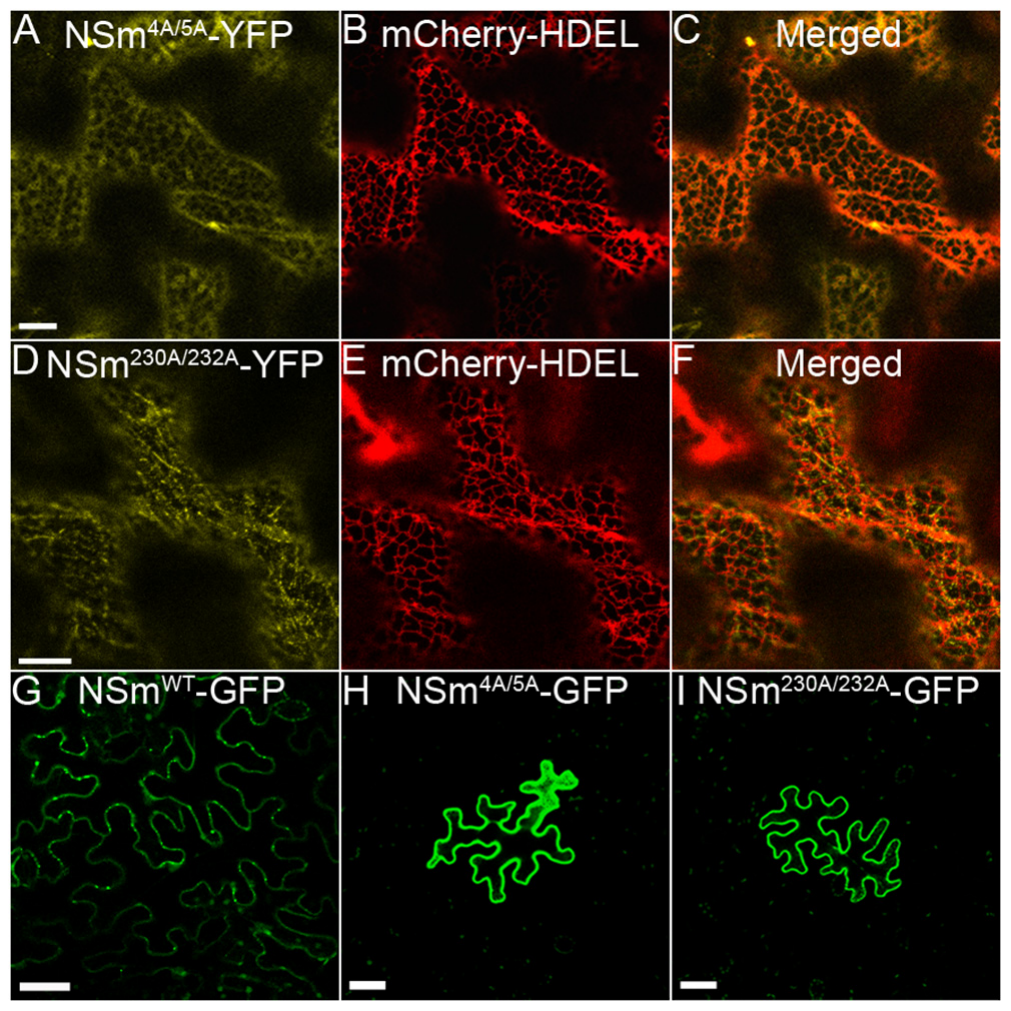
Mutations in NSm that blocked correct ER sorting or altered sorting to other subcellular localization inhibits NSm cell-to-cell movement in leaf epidermis of *N*. *benthamiana*. (A-C) Subcellular localization of NSm^4A/5A^ mutant in epidermal cell. ER marker mCherry-HDEL was used for colocalization analysis. Bar, 10 μm. (D-F) Colocalization analysis of NSm^230A/232A^ mutant with ER labeled by mCherry-HDEL. Bar, 10 μm. (G-I) Cell-to-cell movement analysis of NSm^4A/5A^ (H) and NSm^230A/232A^ (I) mutants in *N*. *benthamiana* after bombardment. NSm^WT^ (G) was used as a positive control. Bar, 50 μm.

Our confocal colocalization analysis showed that NSm formed punctuate structures on the ER (Fig 2A–2C). We reasoned that NSm might contain specific signals for sorting it to the ER. Because di-acid or di-hydrophobic motifs have been shown to play critical roles in ER sorting [77,78], we generated two NSm mutants with alanine substitutions at di-hydrophobic and di-acid amino acids 4–5 (FF) and 230–232 (DKD). We agroinfiltrated *N*. *benthamiana* leaves to transiently express these two mutants, and then analyzed their subcellular localization by confocal microscopy. Colocalization analysis showed that mutant NSm^4A/5A^ formed a smooth structure that aligned well with the ER membrane, but no longer formed punctate structures (Fig 5A–5C), indicating that the mutations blocked NSm sorting to the cortical ER. Mutant NSm^230A/232A^ was present as punctate structures in the cells, but was not localized to the ER (Fig 5D–5F), indicating that it was mis-sorted to other subcellular localizations. We also used sucrose gradient fractionation with and without MgCl_2_ to identify the cellular fraction that contained the mutant NSm^4A/5A^ and NSm^230A/232A^. The immunoblot of gradient fraction showed that NSm^4A/5A^ was distributed mainly in fractions 6–8 with MgCl_2_ and 5–7 without MgCl_2_ (S5B Fig), whereas NSm^230A/232A^ was distributed in fractions 2–4 whether MgCl_2_ was present or not (S5C Fig). As shown in above, in the absence of MgCl_2_, ribosomes became dissociated from the ER, and the NSm^WT^ had the same shift as the ER marker in the gradient (Fig 2J). However, neither NSm^4A/5A^ or NSm^230A/232A^ had the same shift as the NSm^WT^ (S5 Fig). These results suggest that the subcellular localization of the two NSm mutants differed from the ER location of the NSm^WT^.

Via biolistic bombardment, mutants NSm^4A/5A^ and NSm^230A/232A^ were then expressed separately in single cells of *N*. *benthamiana* leaves. Neither NSm^4A/5A^ nor NSm^230A/232A^ could move cell to cell (Fig 5G–5I and Table 1). Thus, sorting of NSm to particular sites on the ER is necessary for its intercellular trafficking.

### Pharmacological disruption of the ER network inhibits NSm cell-to-cell trafficking

If NSm trafficks along the ER membranes, disruption of the ER network should negatively affect this trafficking. We thus tested the effect of brefeldin A (BFA), a pharmacological drug that at high concentrations can disrupt the integrity of the ER network [79,80], on the structural integrity of the ER network in *N*. *benthamiana* leaves expressing mCherry-HDEL. At 3 h post-treatment with 20 μg/mL BFA, the sheet structure of the ER clearly changed (S6A and S6B Fig). Within 6–12 h post-treatment, the ER network was severely disrupted (S6C–S6F Fig).

To test if the disruption of ER membrane by BFA treatment affects cell-to-cell movement of NSm, the bombarded leaf tissues of *N*. *benthamiana* transiently expressing NSm-GFP were treated with 20 μg/mL of BFA or DMSO (as a control) 6 h post bombardment for 12 h, and checked for cell-to-cell trafficking of NSm-GFP by confocal microscopy. As shown in Table 2, 37.9% of the foci showed multicellular fluorescence from the NSm-GFP fusion protein in DMSO-treated control leaf tissues, in contract to a much lower percentage (10.9%) of such foci observed in BFA-treated leaves. Thus, BFA treatment significantly inhibited cell-to-cell trafficking of NSm-GFP. To test whether this inhibition is the result of a general disruption of transport through PD, we assayed cell-to-cell trafficking of GFP, which moves freely between leaf epidermal cells [81,82]. As shown in Table 2, cell-to-cell trafficking of GFP, which most likely occurred through the microchannels formed between the plasma membrane and the ER with PD, was not affected by BFA treatment in comparison with the DMSO treatment (Table 2). Thus, BFA treatment did not affect general transport through PD, but specifically disrupted cell-to-cell trafficking of NSm.

**Table 2:** BFA disruption of the interconnected ER network inhibited cell-to-cell movement of TSWV NSm in *Nicotiana benthamiana*.

Bombarded plasmid	Treatment	Total foci	Number of total signal clusters (Percentage of total)				
			1 cell/cluster	2 cells/cluster	3 cells/cluster	≥4 cells/cluster	*P*-value ^b^
**NSm-GFP**	DMSO	58	36 (62.1**%**) ^a^	6 (10.3**%**)	6 (10.3**%**)	10 (17.3**%**)	
	BFA ^c^	55	49 (89.1**%**)	4 (7.3**%**)	2 (3.6**%**)	0	<0.001
**GFP**	DMSO	136	118 (86.8**%**)	17 (12.5**%**)	1 (0.7**%**)	0	
	BFA	202	180 (89.1**%**)	20 (9.9**%**)	2 (1.0**%**)	0	>0.05

^a^ Signal clusters comprise fluorescent cells showing the presence of GFP or GFP fusion protein.

^b^ *P*-values were calculated using the unpaired two tailed Student *t*-test.

^c^ BFA was used at 20 μg/mL.

These combined results provide pharmacological evidence that NSm trafficked along the ER membrane, rather than via the cytoplasmic channels among cells through PD.

### The ER-to-Golgi secretion pathway and cytoskeleton transport systems are not involved in intercellular movement of TSWV NSm

Because BFA at low concentrations can block transport from the ER to the Golgi apparatus [83], we used 2.5 μg/mL BFA to examine whether the ER-to-Golgi secretion pathway is involved in the cell-to-cell movement of NSm-GFP. Although sufficient to cause the Golgi marker Man49-GFP to retreat back into the ER (S7A–S7F Fig), 2.5 μg/mL BFA had no effect on cell-to-cell movement of NSm-GFP after biolistic bombardment (S4 Table), strongly suggesting that NSm does not enter the ER-to-Golgi early secretion pathway.

Next, we investigated the contribution of cytoskeleton transport components, microfilaments, myosin motors and microtubules, to the intercellular movement of TSWV NSm. To examine the involvement of microfilaments, we treated *N*. *benthamiana* leaves expressing an actin marker with 5 μM latrunculin B (LatB), which disrupted the actin filaments within 5 h (S8A–S8F Fig). However, treatments with LatB longer than 6 h also disrupted the ER membrane structure (S8G–S8I Fig). Thus, we chose another strategy to conduct this experiment. In the time course of cell-to-cell movement established for NSm-GFP, the NSm-GFP moved intercellularly 16.3% of the initial foci expressing fusion proteins by 10 h post bombardment and 34.5% of the foci by 15 h, indicating approximately 18% of the foci underwent cell-to-cell movement between 10 and 15 h. This time frame is thus appropriate for testing the effect of LatB. By adding LatB at 10 h, we could observe the effect of the drug on NSm intercellular movement at 15 h before LatB affects ER structure. As shown in S4 Table, cell-to-cell trafficking of NSm-GFP in the LatB-treated leaf cells did not differ significantly from that in the DMSO-treated control, suggesting that actin filaments are not involved in NSm movement.

We then investigated the contribution of myosin motors to NSm movement by expressing the Golgi marker in *N*. *benthamiana* leaves and treated them with BDM, a myosin inhibitor [84]. We found that 100 mM 2,3-butanedione monoxime (BDM) was sufficient to inhibit intracellular movement of the Golgi bodies (S1 Movie) within 6 h without any significant disturbance of ER structure (S9A–S9F Fig) as seen with confocal microscopy. We thus treated *N*. *benthamiana* leaves bombarded to express NSm-GFP for 6 h with 100 mM of BDM or PBS buffer (as a control). At 5 h post treatment, we checked for cell-to-cell trafficking of NSm-GFP. As shown in S4 Table, there was no significant difference between the BDM-treated and PBS-treated leaf cells in cell-to-cell trafficking of NSm-GFP, suggesting that myosin motors are not involved in the intercellular movement of NSm.

Finally, we investigated the contribution of microtubules to NSm movement. We previously reported that 20 μM oryzalin efficiently disrupts the microtubule filaments [85], and it had no effect on ER membrane structure within 6 h (S9G–S9L Fig). We then delivered NSm-GFP into *N*. *benthamiana* leaf cells via biolistic bombardment. After 10 h, we treated the bombarded leaves with 20 μM of oryzalin or DMSO. When we checked for cell-to-cell trafficking of NSm-GFP 5 h later, we did not observe any significant differences after the oryzalin and control treatments in the cell-to-cell movement of NSm-GFP (S4 Table), suggesting that microtubules are not involved in NSm movement.

Taken together, these data suggest that the ER-to-Golgi secretion pathway and cytoskeleton transport systems are not involved in the intercellular movement of NSm.

### Cell-to-cell trafficking of NSm is significantly reduced in an unbranched ER network mutant of *Arabidopsis thaliana*

We finally tested the ER-based NSm cell-to-cell trafficking hypothesis and its biological significance by a genetic approach. The ER network is formed by homotypic fusion of membrane tubules. In mammalian cells, a class of dynamin-like, membrane-bound GTPases called atlastins are involved in the generation of the tubular ER network [86,87]. In plants, RHD3 (ROOT HAIR DEFECTIVE 3), an analogue of the mammalian atlastin, mediates the generation of the tubular ER network. Knockout of the *RHD3* gene leads to a nonbranched ER network in *Arabidopsis thaliana* [88,89].

**Fig 6:**
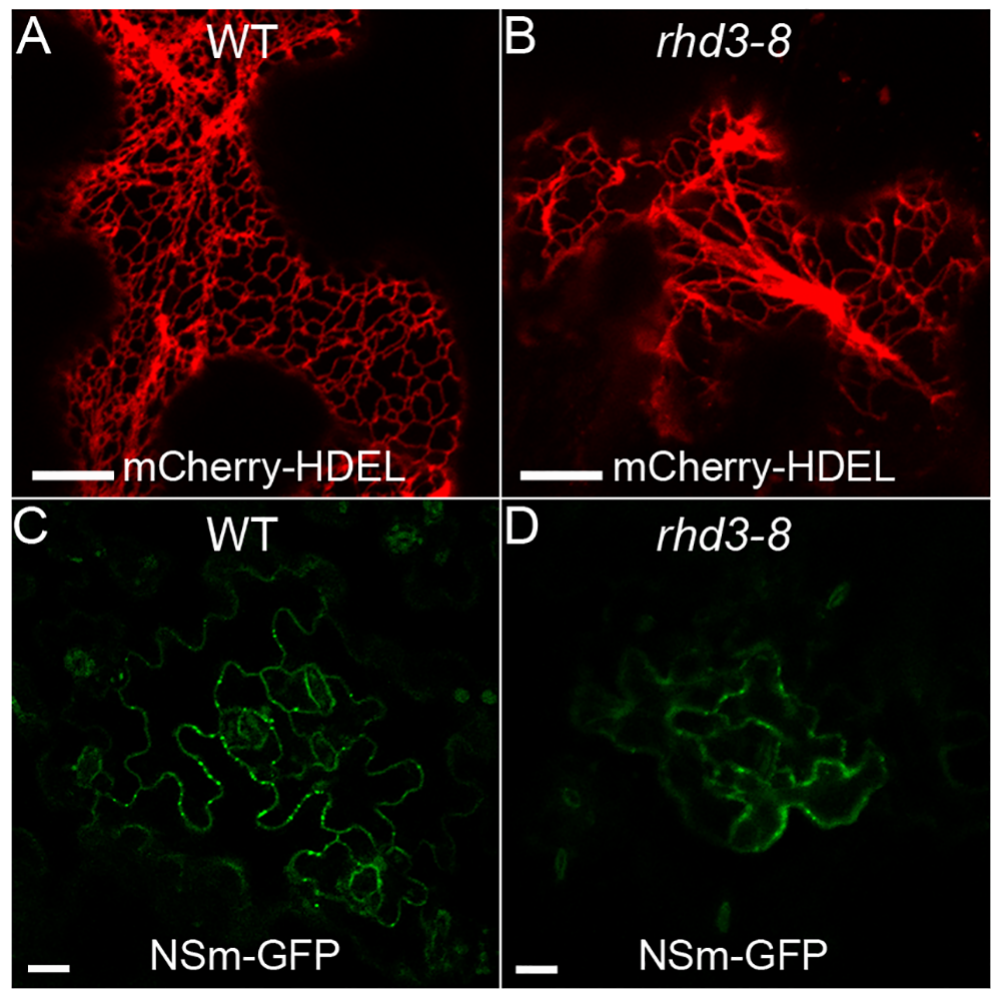
Cell-to-cell trafficking of NSm is significantly reduced in the nonbranched ER network of the *rhd3* mutant of *A*. *thaliana* compared with the Col-0 wild-type (WT). (A and B) Morphology of the ER network labeled by mCherry-HDEL in WT (A) and *rhd3-8* (B), respectively. Bar, 10 μm. (C and D) Comparison of intercellular movement of NSm-GFP in WT and *rhd3-8* plants after bombardment. Bar, 20 μm.

Using the Arabidopsis *rhd3* mutant to investigate whether the altered ER network structure affects cell-to-cell trafficking of NSm, we first confirmed the altered morphology of the ER network in the *rhd3-8* mutant line. A nonbranched ER network labeled by mCherry-HDEL was clearly observed in the *rhd3-8* mutant but not in the wild-type (WT) Col-0 (Fig 6A and 6B). We then used biolistic bombardment to produce NSm-GFP in leaves of the WT and the *rhd3-8* mutant plants. As shown in Fig 6C, 6D and Table 3, cell-to-cell trafficking of NSm was significantly reduced in the *rhd3-8* mutant, compared with that in the WT plants. Specifically, 96 of the 178 (53.9%) fluorescent foci showed NSm trafficking in WT plants, whereas only 60 of the 171 (35.1%) loci did in the *rhd3-8* mutant at 21 h post-bombardment. Furthermore, NSm-GFP moved to 4–6 cells in WT plants in contrast to only 2–4 cells in the *rhd3-8* mutant plants (Table 3). Importantly, cell-to-cell diffusion of GFP did not differ significantly between WT and *rhd3-8* plants (Table 3), indicating that the altered ER structure did not impact general transport through PD microchannels.

**Table 3:** Cell-to-cell movement of TSWV NSm in WT (Col-0) and *rhd3* mutant of *Arabidopsis thaliana* after bombardment.

Bombarded plasmid	Plant	Total foci	Number of signal clusters (Percentage of total)				
			1 cell/cluster	2 cells/cluster	3 cells/cluster	≥ 4 cells/cluster	*P*-value ^b^
**NSm-GFP**	WT	178	82 (46.1**%**) ^a^	28 (15.7**%**)	30 (16.9**%**)	38 (21.3**%**)	
	*rhd3-8*	171	111 (64.9**%**)	35 (20.5**%**)	17 (9.9**%**)	8 (4.7**%**)	<0.001
**GFP**	WT	81	61 (75.3**%**)	12 (14.8**%**)	5 (6.2**%**)	3 (3.7**%**)	
	*rhd3-8*	109	85 (78**%**)	13 (11.9**%**)	5 (4.6**%**)	6 (5.5**%**)	>0.05

^a^ Signal clusters comprise fluorescent cells showing presence of GFP or GFP fusion protein.

^b^ *P*-values were calculated using unpaired two-tailed Student *t*-test.

### Viral systemic infection is significantly delayed in the nonbranched ER network mutant of *A*. *thaliana*

**Fig 7:**
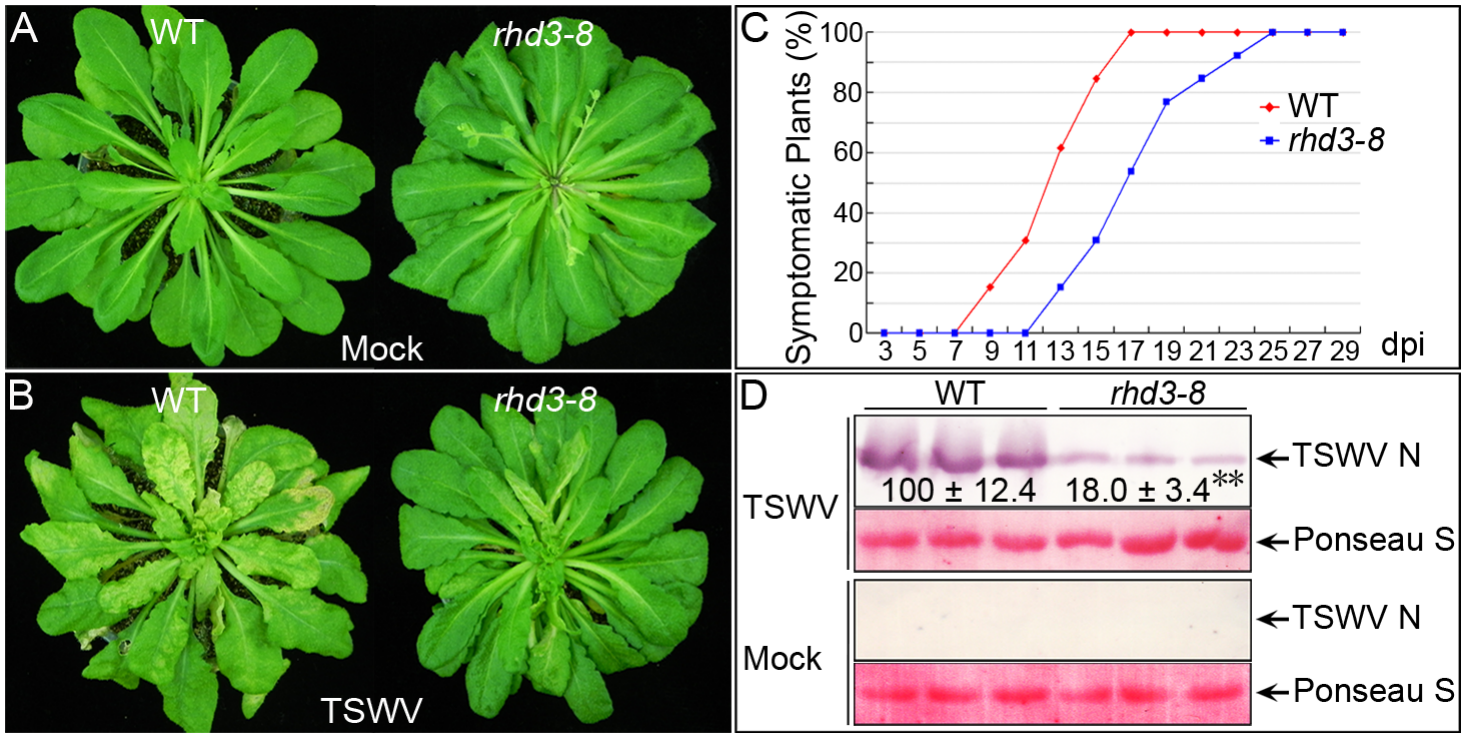
Viral systemic infection is significantly delayed in the nonbranched ER network *rhd3* mutant of *A*. *thaliana* compared with the Col-0 wild-type (WT). (A and B) Symptoms of WT and *rhd3-8* plants inoculated with mock (A) or TSWV (B) at 15 dpi. (C) Disease development was delayed in *rhd3-8* mutant compared with WT after inoculation with TSWV. (D) Immunoblots of extracts from leaves of WT and *rhd3-8* plants after systemic infection with TSWV and probing with monoclonal antibodies against TSWV nucleocapsid at 15 dpi. Protein loading is indicated by Ponceau S staining. The accumulation of TSWV in WT and *rhd3-8* plants was quantified.

Next, we analyzed TSWV infection in WT and *rhd3* mutant plants by mechanically inoculating leaves of these plants with equal amounts of TSWV virions followed by monitoring disease development and viral accumulation over time. As shown in Fig 7A–7C and S5 Table, TSWV disease development was much slower in the infected *rhd3-8* mutant plants than in the infected WT plants. In contrast to the 100% infection and typical cell death symptoms at ∼15 days post-inoculation in the WT plants, disease symptoms were delayed by 3–5 days and attenuated in the *rhd3* mutant plants (Fig 7A–7C and S5 Table). Immunoblots of viral accumulation in systemically infected leaves, probed with a monoclonal antibody against the TSWV nucleocapsid, at 15 days postinoculation showed that TSWV accumulation was significantly lower in the *rhd3-8* mutant plants than in the WT plants (Fig 7D).

To determine whether the reduced virus infection resulted from delayed cell-to-cell movement or from reduced viral replication or both, protoplasts were isolated from WT and *rhd3-8* mutant and transfected with purified particles of TSWV. At 24 h post transfection, protoplasts were harvested, and the accumulation of viral genomic RNAs or proteins of TSWV was examined by quantitative real-time RT-PCR or immunoblot. As shown in S10A Fig, replication of the TSWV M (left panel) or S (right panel) RNA segment in *rhd3-8* was comparable to that in WT. The expression level of TSWV nonstructural protein NSm (right upper panel) or NSs (right middle panel), which was not present in the purified viral particles, in the *rhd3-8* mutant was also similar to that in WT (S10B Fig), suggesting that the reduced virus infection in *rhd3-8* is due to delayed cell-to-cell movement of NSm.

Together, these results provided genetic evidence to support the hypothesis that TSWV NSm employs the ER membrane network as a route for cell-to-cell trafficking. Furthermore, the results indicate that this route is important for viral infection.

## Discussion

Previous studies have demonstrated that different plant viruses may use different host cell transport machineries to move from one cell to another through PD [13–17,22]. In the present study, we confirmed that TSWV NSm is physically associated with the ER membrane, revealed that the NSm moves between cells through the PD, and evaluated the contribution of the ER membrane transport system to the intercellular movement of NSm and TSWV. We have obtained comprehensive biochemical, cellular and genetic evidence that the ER membrane transport system is critical for cell-to-cell trafficking of NSm and the virus.

A recent study suggests that NSm from four tospoviruses are peripherally associated with membranes [58], based on results from a treatment with 7 M urea. We observed that TSWV NSm is still tightly associated with the membrane after treatment with 4 M urea, consistent with a previous suggestion that GNRV NSm is also tightly associated with or even integrated into the membrane [90]. Although 7 M urea is a strong treatment that should release all peripheral proteins from the membrane [65], it only extracted half of the NSm molecules from the membrane fraction. Our *in vitro* membrane insertion experiment clearly showed that TSWV NSm HRs can integrate to a certain extent into the membrane. Triton X-114 partition analysis further showed that NSm from transient expression or virus infection is physically and tightly associated with membrane. These results suggest that TSWV NSm is not a peripheral membrane protein as recently reported [58]. However, the strength of the membrane association with NSm is not as strong as for a canonical integral membrane protein that span the membrane. The 7 M urea treatment extracted more NSm protein molecules than it did for PVX TGB2 and TGB3; the insertion capability of NSm was not as good as that of previously reported, membrane-spanning proteins [64,67,69]. All these data suggest that the membrane insertion capability of NSm lies between the peripheral membrane protein and the integral membrane protein. The new conclusion that could fit all previous data and our results in this study is that NSm strongly and physically associates with the membrane, but probably does not span the lipid bilayer. Thus, tospovirus NSm may represent a new subcategory of movement protein with a distinct membrane association property in 30K superfamly.

NSm-GFP (60.9 kDa) expressed in a single cell of *N*. *benthamiana* moved efficiently from one cell to another, which is supported by the fact that TSWV NSm can increase the size-exclusion limit (SEL) of the PD [50,54]. However, the dilated SEL of the PD itself is not sufficient to support cell-to-cell movement of cytoplasmic proteins such as GFP-GFP (54.0 kDa) in the presence of NSm. As we mentioned earlier, NSm is physically and tightly associated with the ER membrane (Figs 1 and 2). Mutations in NSm that disrupted its association with the ER membrane or caused mis-sorting to other subcellular localization inhibited cell-to-cell trafficking, suggesting that targeting onto the ER is a critical step for cell-to-cell movement. We observed that NSm forms inclusion bodies on the ER. NSm predominantly targets the ER where it likely accumulates within the plant cell. The NSm inclusions were easily detected when NSm was transiently expressed after agroinfiltration. However, the NSm inclusion bodies were not observed in cells from which NSm trafficked to adjacent cells in the biolistic bombardment assay, suggesting that the inclusion bodies probably are due to NSm accumulation on the ER. Although we do not yet know whether NSm inclusion bodies have any other biological function for TSWV, these inclusion bodies are a reliable indicator of the ER targeting of NSm. The NSm^4A/5A^ mutant that did not induce ER inclusions was not in the same sucrose gradient fraction as the ER, and it was defective in cell-to-cell trafficking, further supporting that targeting the ER is an important step for its intercellular movement.

The plant ER is interconnected among cells via the ER desmotubule in the plasmodesma of the cell wall [18,59]. Pharmacological disruption or a genetic defect in the ER network inhibited NSm-GFP trafficking but not GFP diffusion, which occurs through the cytoplasmic channels. The defect in the ER network strongly delayed cell-to-cell spread of TSWV. These results demonstrated that an intact ER membrane transport system is critical for intercellular movement of viral MP and TSWV. The plasmolysis assay showed that NSm remained on the PD after the cytoplasmic ER was separated from the cell wall (Fig 2G–2I). The PD localization of NSm and its physical association with the ER strongly suggest that NSm and TSWV may move between cells through PD via the desmotubule ER. Compared with the discrete ER confined within each cell in animals, the plant ER forms a continuous membrane network throughout the entire plant [18,59]. Such a unique ER membrane network structure in plant provides an effective continuous transport route to deliver NSm and TSWV to neighboring cells or to remote growing tissues.

Although molecular genetics of the animal-infecting members of the *Bunyaviridae* have been facilitated by the rescue of infectious cDNA clones, a reverse genetics system remains elusive for all members of the multipartite negative-strand plant RNA viruses. Despite discovering the NSm mutant that is defective in its ER association or ER sorting, without infectious cDNA clones of TSWV, it is difficult for us to use this system to evaluate the contribution of the ER-membrane association of NSm to the intercellular trafficking of the virus. We have also tested the BFA effects on the intercellular movement of TSWV; however, a high concentration of BFA caused severe cell death in infiltrated leaves of *N*. *benthamiana* or *N*. *tobacum* cv. Samsun after 30 h of treatment. Chemical treatments have been widely used in the studies of virus movement [28,37,85]. But as we mentioned in the results, prolonged incubation with chemicals has nonspecific effects on the plant, especially when drugs must be used for days to see any effects on virus infection and thus greatly interferes with the interpretation of results. Thus, with this system, evaluating the contribution of the ER membrane network to intercellular movement of virus is also difficult. We were finally able to find an *rhd3* mutant that is defective in the generation of the tubular ER network. This mutant with nonbranched ER gives a clean background and consistent outcome and provides us a powerful genetic system to investigate the contribution of the ER membrane network to the intercellular movement of TSWV. By using this system, we were able to identify that virus infection was strongly delayed in the *rhd3* mutant. The protoplast assay further revealed that the reduced viral infection was not due to defective replication. Thus, we established that the ER membrane transport system is important for TSWV to establish infection of the plant.

Although NSm accumulated on the ER membrane, we found that the ER-to-Golgi secretion pathway is not involved in NSm movement. Subcellular localization showed that NSm was not exported onto the Golgi body. Treatment with a low concentration of BFA that causes the redistribution of a Golgi marker back to the ER did not affect the intercellular movement of NSm. Although cytoskeleton transport systems are important for the intercellular movement of many MPs and viruses [28,30,36–40]. Our pharmaceutical treatment analysis showed that microfilaments, myosin motors and microtubules are not involved in the cell-to-cell movement of TSWV NSm. However, we showed previously that TSWV nucleocapsids, the major component of the VRC, move intracellularly along the ER and actin microfilaments [85]. The actin microfilaments and myosin XI-K were found to be responsible for the intracellular movement of the TSWV nucleocapsid and for the virus to infect the plant [85]. Studies of the classical virus TMV have demonstrated that microfilaments and myosin motors are important for intracellular movement of viral VRC [40,91], whereas the ER membrane to which the VRC and MP are anchored have been proposed to play essential roles in the cell-to-cell movement of the MP and virus by diffusing between cells through the PD [92]. In the present study, we demonstrated that the ER membrane transport system is critical for intercellular trafficking of the NSm MP and TSWV. Based on our previous and current findings, a new picture is emerging for TSWV that microfilaments and myosins are involved in the intracellular trafficking of viral VRC, whereas the ER membrane transport system, where both NSm and VRC associate, provides a critical direct route for NSm and the virus to move from cell to cell. Tospovirus NSm is predominantly membrane associated [49,51,58]. The VRC of *Fig mosaic virus*, another multipartite negative-strand plant RNA virus, was also found to traffic intracellularly along the ER and actin microfilaments [80]; however, little has been known about the mechanism of intercellular movement of multipartite negative-strand RNA plant viruses. Our findings have important new implications to guide future mechanistic studies on intercellular trafficking of tospoviruses and other multipartite negative-strand plant RNA viruses.

In summary, we showed in this study that the ER membrane transport system is important for the intercellular movement of NSm and TSWV. Because NSm localized on both the ER and PD, the virus likely recruits essential host factors to facilitate its cell-to-cell movement via the desmotubule ER. Identifying the host proteins within the ER membrane transport system that interact with the viral proteins and enable virus movement will be the focus of our future investigations.

## Materials and Methods

### Plasmid constructs and organelle markers

The NSm gene was amplified from total RNA isolated from TSWV YN-infected tomato by RT-PCR. The ER markers mCherry-HDEL and YFP-HDEL, the Golgi markers Man49-mCherry and Man49-GFP [93] were obtained from the Arabidopsis Biological Resource Center (ABRC). H2B-mRFP was constructed by fusing H2B and mRFP by overlap PCR. All constructs and primers used in this study are listed in S6 Table.

For expression of Lep-derived constructs harboring HR from NSm protein, the Lep sequence carried one glycosylation acceptor site in positions 3–5 of an extended sequence of 24 residues previously described [70]. Oligonucleotides encoding the HR1 and HR2 sequences were introduced replacing H2 TM segment from Lep. Tested sequences were constructed using two double-stranded optimized oligonucleotides with 5′ phosphorylated overlapping overhangs at the ends. Pairs of complementary oligonucleotides were first annealed at 85°C for 10 min followed by slow cooling to 30°C, after which the two annealed double-stranded oligonucleotides were mixed, incubated at 65°C for 5 min, cooled slowly to room temperature and ligated into the vector. NSm-derived segment inserts were confirmed by sequencing of plasmid DNA.

### Plant material, transient expression and virus inoculation

Six- to eight-week-old plants of *N*. *benthamiana* or *A*. *thaliana* were used for all transient expression analyses and virus inoculations. Homozygous seeds of the *rhd3-8* mutant of *A*. *thaliana* (SALK_025215) were a gift from Dr. Junjie Hu (Nankai University, Tianjin, P. R. China). TSWV was maintained in *N*. *rustica* plants, and sap from fresh systemically infected leaves were used as inocula. *Agrobacterium tumefaciens* cells (GV3101) containing various NSm constructs and organelle markers were treated with infiltration buffer (10 mM MgCl_2_, 10 mM MES, pH 5.9, and 150 μM acetosyringone) for 3 h at room temperature before infiltration (OD600 = 0.2) of the abaxial side of *N*. *benthamiana* leaves. All agroinfiltrated or virus-inoculated plants were grown in growth chambers (model GXZ500D, Jiangnan Motor Factory, Ningbo, P. R. China) at 25°C with 16 h light/8 h dark for *N*. *benthamiana* and 8 h light/16 h dark for *A*. *thaliana*. Plasmolysis was carried out by infiltrating leaves with 10% NaCl; cells were immediately examined microscopically for the separation of the plasma membrane from the cell wall.

### *In vitro* translation and membrane insertion assay

*In vitro* translation and membrane insertion assays for NSm HRs were done in the presence of reticulocyte lysate, [^35^S]Met/Cys, and dog pancreas microsomes as described previously [69]. Translation membranes were collected by ultracentrifugation and analyzed by SDS-polyacrylamide gel electrophoresis (SDS-PAGE); gels were visualized on a Fuji FLA3000 phosphorimager with ImageGauge software. For the proteinase K protection assay, the translation mixture was digested with proteinase K for 40 min on ice; the reaction was stopped by adding 1 mM phenylmethylsulfonyl fluoride (PMSF) before SDS-PAGE analysis.

### Subcellular fractionation and chemical treatment

Fractionation was done as described by Peremyslov *et al*. [75]. P30 membrane fractions were treated with lysis buffer (20 mM HEPES pH 6.8, 150 mM potassium acetate, 250 mM mannitol, 1 mM MgCl_2_), 0.1 M Na_2_CO_3_, 1 M KCl, 4 M urea, 7 M urea or 1% Triton X-114 as described by Peiró *et al*. [66]. Chemically treated samples were fractionated into the P30 pellet and S30 supernatant. The fractions were analyzed by immunoblotting.

### Sucrose gradient centrifugation

Sucrose gradient fractionation in the presence or the absence of MgCl_2_ was performed as described by Peremyslov *et al*. [75]. NSm-expressing leaves were ground in lysis buffer. The P30 pellet, prepared as described above, was loaded on the top of 20 to 60% linear sucrose gradients prepared with lysis buffer. Samples were centrifuged for 16 h at 100,000 × *g* at 4°C, and 14 fractions of 1.2 mL each were collected from top to bottom. Each fraction was precipitated with trichloroacetic acid (TCA) and analyzed by immunoblotting.

### Confocal laser scanning microscopy and co-localization assays

Confocal images were captured with an inverted Zeiss LSM 710 CLSM and 20× or 63× water immersion objective lenses. YFP and GFP were excited with 488 nm wavelength and emissions at 497–520 nm captured. mRFP and mCherry were excited with 561 nm wavelength and emissions at 585–615 nm captured. For the visualization of chloroplasts, chlorophylls were excited with 488 nm wavelength and emission at 660–720 nm captured. Images were processed using a Zeiss 710 CLSM and Adobe (San Jose, CA, USA) Photoshop.

### Biolistic bombardment and drug treatments

Constructs pRTL2-NSm-GFP, pRTL2-GFP-GFP and pRTL2-GFP were coated with 0.6-μm gold micro-carriers. Leaves from *N*. *benthamiana* or *A*. *thaliana* plants were bombarded using a handheld Helios Gene Gun (Bio-Rad, Hercules, California, USA). Bombarded plants were maintained under the same growth conditions and imaged at various times after bombardment.

For BFA treatment, *N*. *benthamiana* leaves were bombarded with the constructs expressing NSm-GFP or GFP. After 6 h, the bombarded leaves were infiltrated with a low (2.5 μg/mL) or high concentration (20 μg/mL) of BFA (Biyuntian, Haimen, P. R. China). Equivalent dilutions of DMSO were used as controls. After 12 h, cell-to-cell trafficking of NSm-GFP was examined with a CLSM.

For LatB, BDM and oryzalin treatments, *N*. *benthamiana* leaves were bombarded with the construct expressing NSm-GFP. After 10 h, the bombarded leaves were treated with 5 μM LatB (Sigma, Shanghai, China), 100 mM BDM (Sigma) or 20 μM oryzalin (Sigma). Equivalent dilutions of DMSO or PBS buffer were used as controls. After another 5 h, cell-to-cell trafficking of NSm-GFP was examined with a CLSM.

### Inoculation of protoplasts with TSWV virions

TSWV particles were purified as described by Kikkert *et al*. [94]. TSWV virions were isolated at 4°C from systemically infected *N*. *rustica* leaves. The virions were homogenized in sterile double-distilled water and stored at −80°C. Before protoplast inoculation, virions were thawed slowly on ice.

*Arabidopsis* protoplasts were prepared as described by Yoo *et al*. [95] from rosette leaves of 4-week-old *Arabidopsis* plants. Then 0.5–1 × 10^6^ protoplasts were transfected with 2 μg TSWV virions using polyethylene glycol (PEG3350) according to Kikkert *et al*. with minor modifications [94]. At 24 h post inoculation, protoplasts were harvested and analyzed by real-time PCR and protein immunoblotting.

### Quantitative real-time PCR (qRT-PCR)

Total RNA from *Arabidopsis* protoplasts was extracted using an RNAsimple Total RNA kit (Tiangen, Beijing, P. R. China). First-strand cDNA was synthesized using a PrimeScrip RT reagent kit with gDNA eraser (Takara, Dalian, P. R. China), and the cDNA was then amplified using a Power SYBR Green Master Mix (Life Technologies, Carlsbad, California, USA). Primers (S6 Table) specific for the TSWV M segment and S segment were used for quantitative analyses of RNA level of TSWV in protoplasts. The qRT-PCR was performed in an ABI 7500 Real-Time PCR system (Life Technologies). Actin 2 served as an internal control to normalize the RNA levels of target gene between samples using a relative quantification method.

### Western blot analysis

Western blotting was carried out as described previously [85]. Protein samples were separated by electrophoresis in 10% SDS-PAGE and transferred onto a PVDF membrane. The antigens on the PVDF membrane were detected with antibodies against TSWV NSm, N, NSs, Arf1, BiP, PEPC, V-H-ATPase, GFP, or HA, followed by AP-coupled goat anti-mouse IgG (1:10,000 dilution; Sigma) and 5-bromo-4-chloro-3-indolylphosphate/nitroblue tetrazolium (NBT/BCIP) staining (Sangon Biotech, Shanghai, China).

## Supporting Information

S1 Fig Prediction of hydrophobic regions for TSWV NSm. (A) Schematic representation of the NSm protein of TSWV, highlighting the hydrophobic regions (HRs, blue boxes). The HRs comprised aa 127 to 153 (HR1) and 163 to 192 (HR2) as predicted by the ΔG prediction algorithm available at the ΔG prediction server v1.0 (http://dgpred.cbr.su.se/). (B) Hydrophobic profile of NSm generated with MPEx. The red lines show the mean values using a window of 19 residues. The yellow line indicates the predicted hydrophobic regions.
(TIF)

S2 Fig Colocalization of the NSm-YFP inclusion bodies with Golgi (A-C), nuclei (D-F) and chloroplasts (G-I) in leaf epidermis of *N*. *benthamiana*. NSm-YFP fluoresces yellow; the Golgi marker Man49-mCherry, nucleus marker H2B-mRFP and chlorophyll in the chloroplasts fluoresce red. Image was taken at 28 h post infiltration (hpi) with the constructs. Bar, 10 μm.
(TIF)

S3 Fig Cell-to-cell movement of NSm in leaf epidermis of *N*. *benthamiana* after agroinfiltration. (A) Schematic diagram of the plant construct to co-express either NSm-GFP and mCherry-HDEL or GFP-GFP and mCherry-HDEL. (B) Agroinfiltration assay of NSm-GFP to assess cell-to-cell trafficking. *Agrobacterium* containing a construct to co-express either NSm-GFP and mCherry-HDEL (upper panel) or GFP-GFP and mCherry-HDEL (lower panel) was diluted 500 times for expression in a single epidermal cell. Bar, 50 μm.
(TIF)

S4 Fig NSm-GFP moves along the ER membrane network for cell-to-cell transport in leaf epidermis of *N*. *benthamiana* after biolistic bombardment. (A-C) Colocalization of NSm-GFP with the mCherry-HDEL at plane of ER layer in image D at Fig 3. Cell 1, 2 and 3 refers to the initially bombarded cell, second layer of cells and third layer of cells, respectively, where NSm subsequently moved. Bar, 10 μm.
(TIF)

S5 Fig Sucrose density gradient fractionation of the mutant NSm^4A/5A^ and NSm^230A/232A^ in the presence or the absence of MgCl_2_. (A-C) Extracts of plants transiently expressing NSm^4A/5A^ (B) and NSm^230A/232A^ (C) were ultracentrifuged in a 20–60% sucrose gradient in the presence or absence of MgCl_2_. NSm^WT^ (A) was used as a control. Fractions from top to bottom (fraction numbers from 1 to 14) were immunoblotted using anti-NSm antibodies.
(TIF)

S6 Fig Effect of BFA on morphology of ER network. (A-B) ER sheet structure increased after 3-h treatment with 20 μg/mL BFA. (C-F) The ER network was severely disrupted after treatment with 20 μg/mL BFA at 6 h (C and D) or 12 h (E and F). *N*. *benthamiana* was agroinfiltrated with the ER marker mCherry-HDEL to label the ER network. The equivalent amount of DMSO was added as a negative control. Bar, 10 μm.
(TIF)

S7 Fig Redistribution of Golgi apparatus into ER after low concentration BFA treatment. (A-F) Effect of DMSO (A-C) or 2.5 μg/mL BFA (D-F) on Golgi bodies marked by Man49-GFP. At 24 h post agroinfiltration, the infiltrated leaf was treated with BFA or DMSO, then examined 12 h later with confocal microscopy. Bar, 10 μm.
(TIF)

S8 Fig Effects of BFA on ER membrane network and actin microfilaments. (A-C) The ER membrane and actin microfilament structure by DMSO control at 7 h post treatments. (D-I) The ER membrane and actin microfilament structure by 5 μM LatB at 5 h (D-F) or 7 h (G-I) post treatments. *N*. *benthamiana* was agroinfiltrated with the mCherry-HDEL and GFP-ABD2-GFP to label the ER network and actin microfilament, respectively. The cells were examined by confocal microscope. Bar, 10 μm.
(TIF)

S9 Fig Effects of BDM and oryzalin on ER membrane network. (A-F) The ER membrane and Golgi bodies structure by PBS control or by 100 mM BDM at 6 h post treatments. (G-L) The ER membrane and microtubule structure by DMSO control or by 20 μM oryzalin at 6 h post treatments. *N*. *benthamiana* was agroinfiltrated with the mCherry-HDEL/YFP-HDEL, Man49-mCherry and mCherry-MAP65-1, respectively, to label the ER network, Golgi bodies and microtubules. The cells were examined by confocal microscope. Bar, 10 μm.
(TIF)

S10 Fig Replication of TSWV in protoplasts isolated from WT or *rhd3* mutant of *Arabidopsis thaliana*. (A) Quantification of the accumulation of TSWV M (left panel) or the S (right panel) RNA segment in protoplasts of WT or *rhd3* mutant by real-time RT-PCR. Primer pairs targeting NSm and NSs, respectively, were used to quantify the replication of the M and the S segment. (B) Expression level of TSWV nonstructural protein NSm (right upper panel) and NSs (right middle panel) in protoplasts of the WT or *rhd3* mutant by immunoblotting. Protoplasts were isolated from fresh leaves of the WT or *rhd3* mutant. Purified TSWV particles or PBS buffer (mock) were used to transfect protoplasts using PEG3350. Samples were collected 24 h after TSWV transfection for qRT-PCR or immunoblotting.
(TIF)

S1 Table Transmembrane (TM) or hydrophobic region (HR) analysis of TSWV NSm using different computational tools. (DOC)

S2 Table Time course analysis of cell-to-cell movement of NSm-GFP in leaf epidermis of *Nicotiana benthamiana* by bombardment. (DOC)

S3 Table Cell-to-cell movement assay for GFP-GFP in leaf epidermis of *Nicotiana benthamiana* in the presence or the absence of NSm. (DOC)

S4 Table Cell-to-cell trafficking of NSm-GFP in *N*. *benthamiana* was not affected by interfering the ER-to-Golgi early secrection pathway or the cytoskeleton transport systems. (DOC)

S5 Table TSWV infection assay on wild-type (WT) and *rhd3-8* mutant plants of *Arabidopsis thaliana* from 7 to 27 days after inoculation (dpi). (DOC)

S6 Table List of primers used in this study. (DOC)

S1 Movie Effect of PBS and 100 mM BDM on myosin motors in *N*. *benthamiana* leaf cells. Leaves of *N*. *benthamiana* were agroinfiltrated with Golgi marker Man49-GFP, then 33 h later infiltrated with PBS or 100 mM BDM. Time-lapse confocal microscopic images were captured 6 h after the start of the drug treatment.
(AVI)
