# Supplementary figures and images for: The ER-Membrane Transport System Is Critical for Intercellular Trafficking of the NSm Movement Protein and Tomato Spotted Wilt Tospovirus
Source: PLoS Pathog. 2016 Feb 10;12(2):e1005443. doi: 10.1371/journal.ppat.1005443 (PMC4749231; doi:10.1371/journal.ppat.1005443)

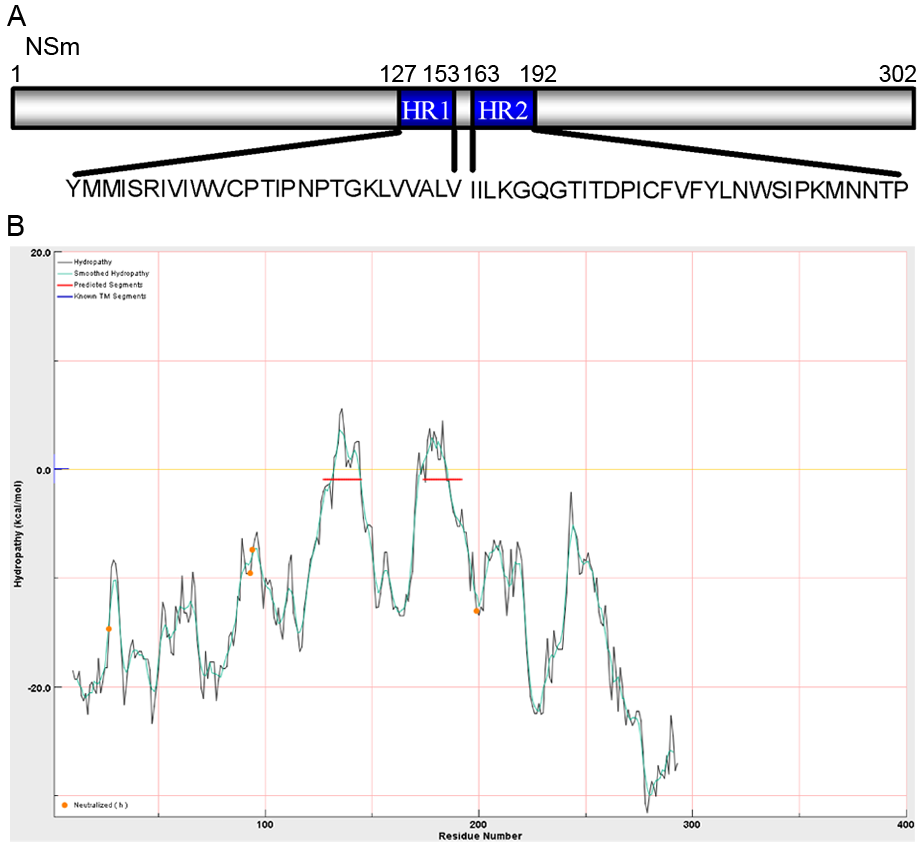

Supplement: S1 Fig — (A) Schematic representation of the NSm protein of TSWV, highlighting the hydrophobic regions (HRs, blue boxes). The HRs comprised aa 127 to 153 (HR1) and 163 to 192 (HR2) as predicted by the ΔG prediction algorithm available at the ΔG prediction server v1.0 (http://dgpred.cbr.su.se/). (B) Hydrophobic profile of NSm generated with MPEx. The red lines show the mean values using a window of 19 residues. The yellow line indicates the predicted hydrophobic regions. (TIF) [file ppat.1005443.s001.tif]

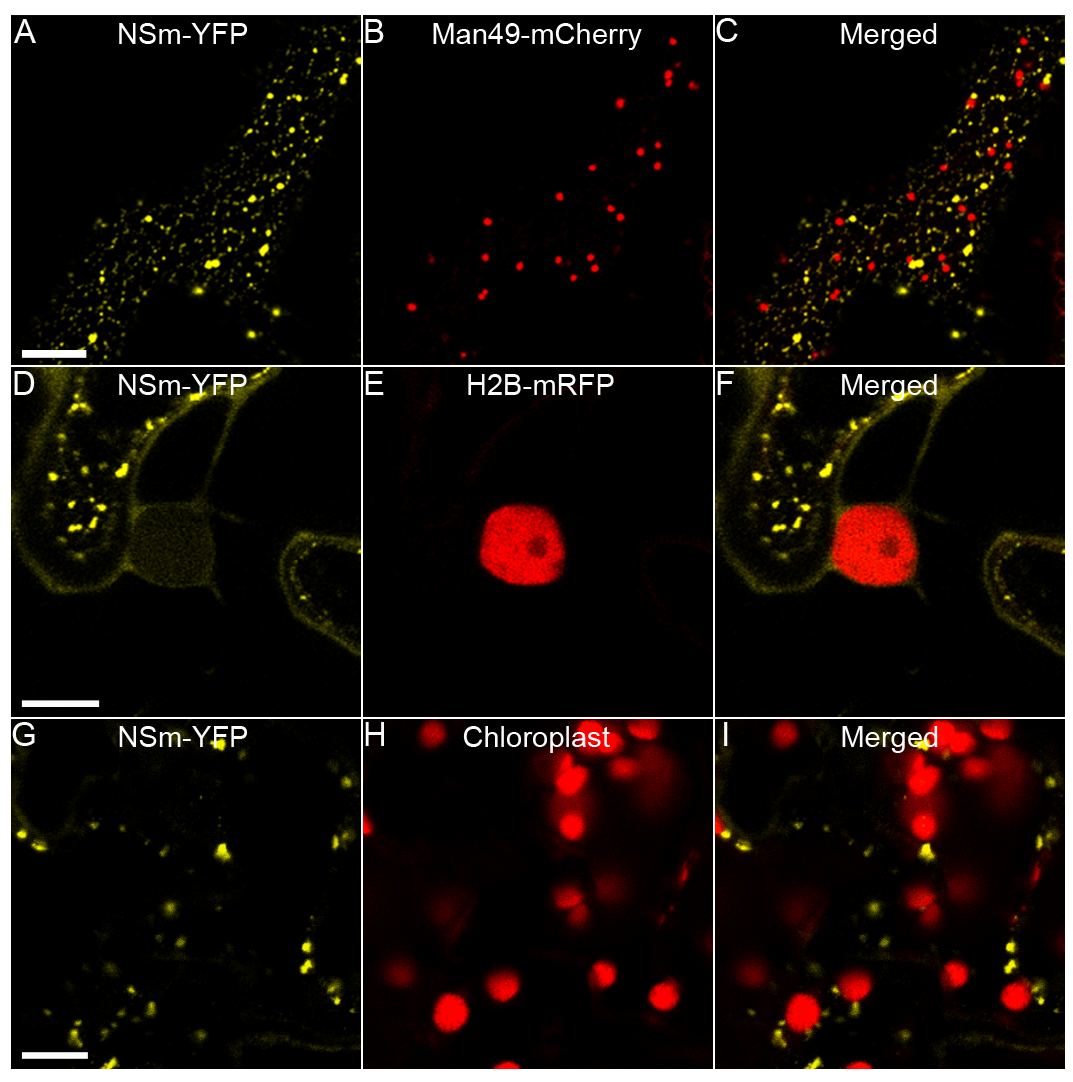

Supplement: S2 Fig — NSm-YFP fluoresces yellow; the Golgi marker Man49-mCherry, nucleus marker H2B-mRFP and chlorophyll in the chloroplasts fluoresce red. Image was taken at 28 h post infiltration (hpi) with the constructs. Bar, 10 μm. (TIF) [file ppat.1005443.s002.tif]

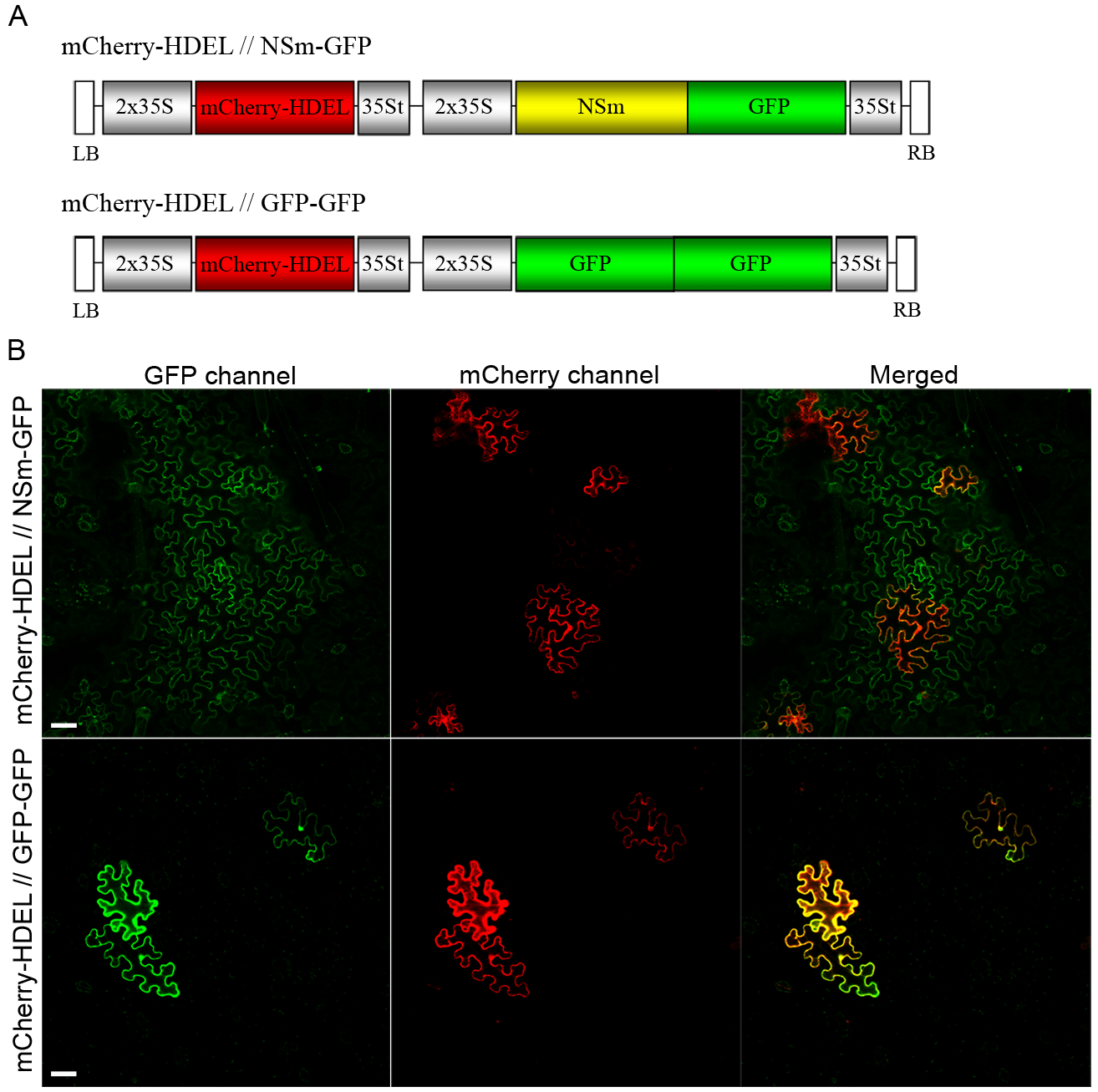

Supplement: S3 Fig — (A) Schematic diagram of the plant construct to co-express either NSm-GFP and mCherry-HDEL or GFP-GFP and mCherry-HDEL. (B) Agroinfiltration assay of NSm-GFP to assess cell-to-cell trafficking. Agrobacterium containing a construct to co-express either NSm-GFP and mCherry-HDEL (upper panel) or GFP-GFP and mCherry-HDEL (lower panel) was diluted 500 times for expression in a single epidermal cell. Bar, 50 μm. (TIF) [file ppat.1005443.s003.tif]

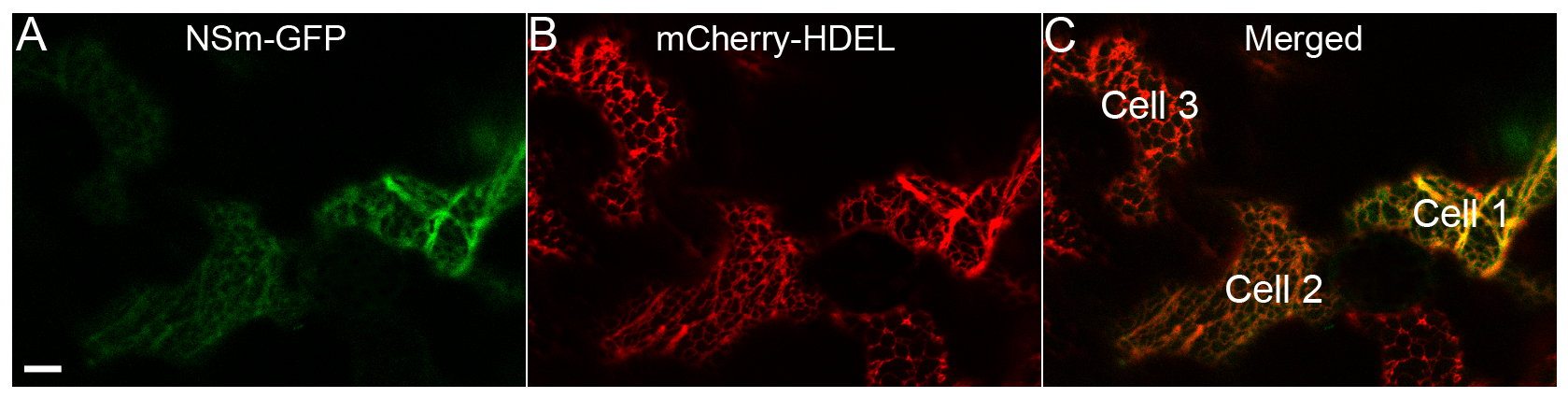

Supplement: S4 Fig — (A-C) Colocalization of NSm-GFP with the mCherry-HDEL at plane of ER layer in image D at Fig 3. Cell 1, 2 and 3 refers to the initially bombarded cell, second layer of cells and third layer of cells, respectively, where NSm subsequently moved. Bar, 10 μm. (TIF) [file ppat.1005443.s004.tif]

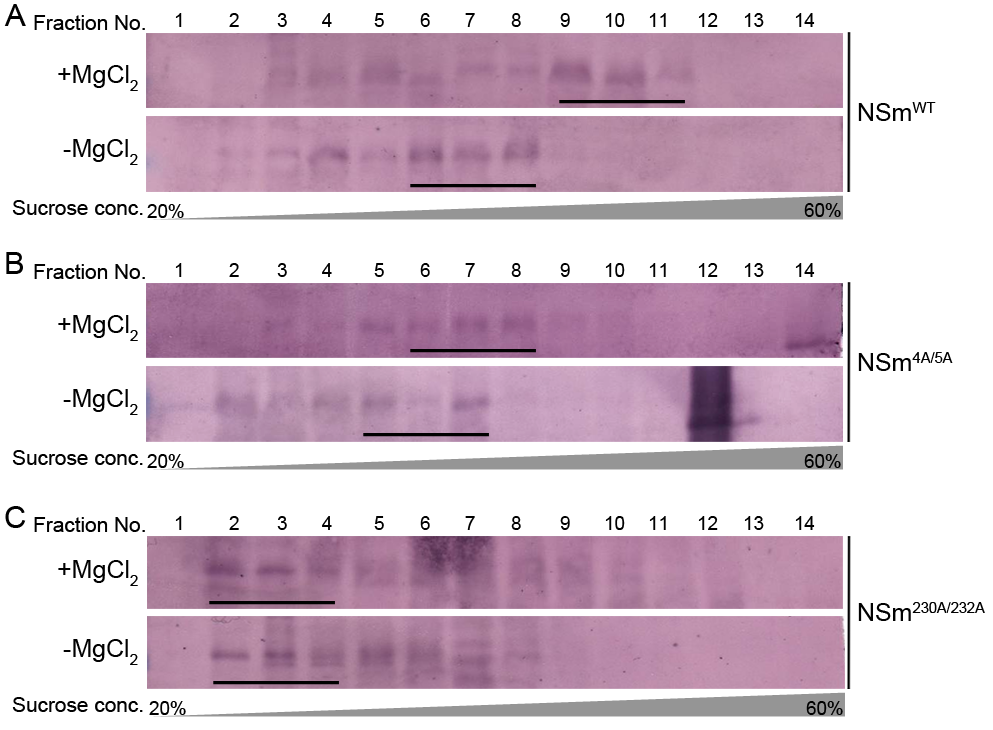

Supplement: S5 Fig — (A-C) Extracts of plants transiently expressing NSm4A/5A (B) and NSm230A/232A (C) were ultracentrifuged in a 20–60% sucrose gradient in the presence or absence of MgCl2. NSmWT (A) was used as a control. Fractions from top to bottom (fraction numbers from 1 to 14) were immunoblotted using anti-NSm antibodies. (TIF) [file ppat.1005443.s005.tif]

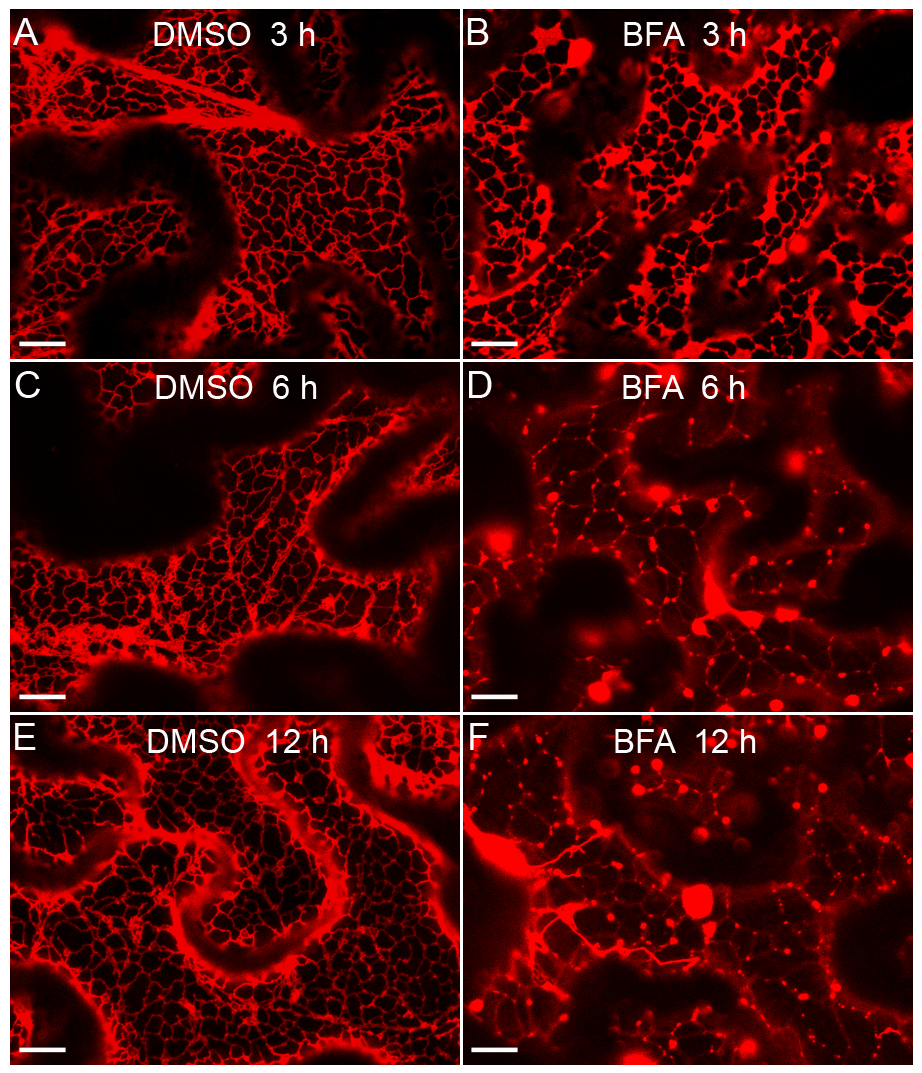

Supplement: S6 Fig — (A-B) ER sheet structure increased after 3-h treatment with 20 μg/mL BFA. (C-F) The ER network was severely disrupted after treatment with 20 μg/mL BFA at 6 h (C and D) or 12 h (E and F). N. benthamiana was agroinfiltrated with the ER marker mCherry-HDEL to label the ER network. The equivalent amount of DMSO was added as a negative control. Bar, 10 μm. (TIF) [file ppat.1005443.s006.tif]

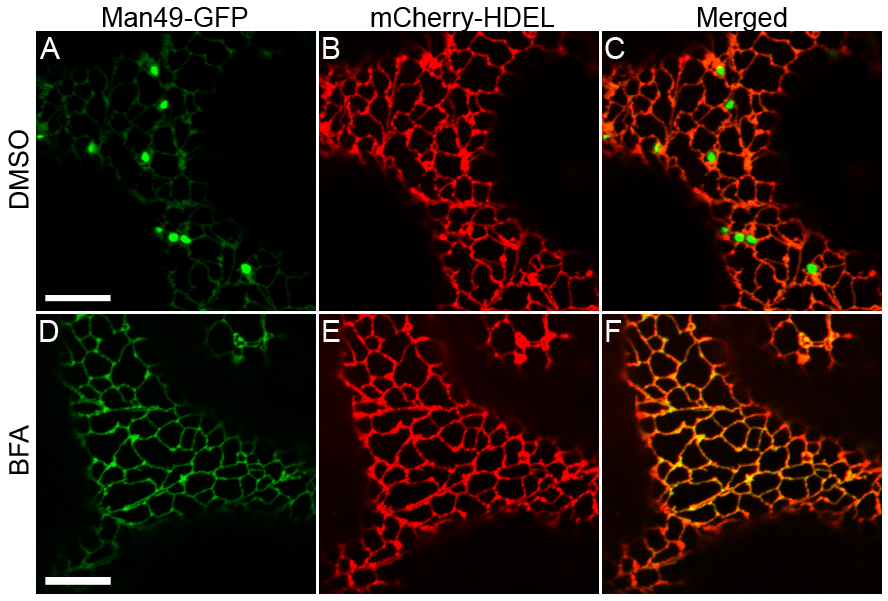

Supplement: S7 Fig — (A-F) Effect of DMSO (A-C) or 2.5 μg/mL BFA (D-F) on Golgi bodies marked by Man49-GFP. At 24 h post agroinfiltration, the infiltrated leaf was treated with BFA or DMSO, then examined 12 h later with confocal microscopy. Bar, 10 μm. (TIF) [file ppat.1005443.s007.tif]

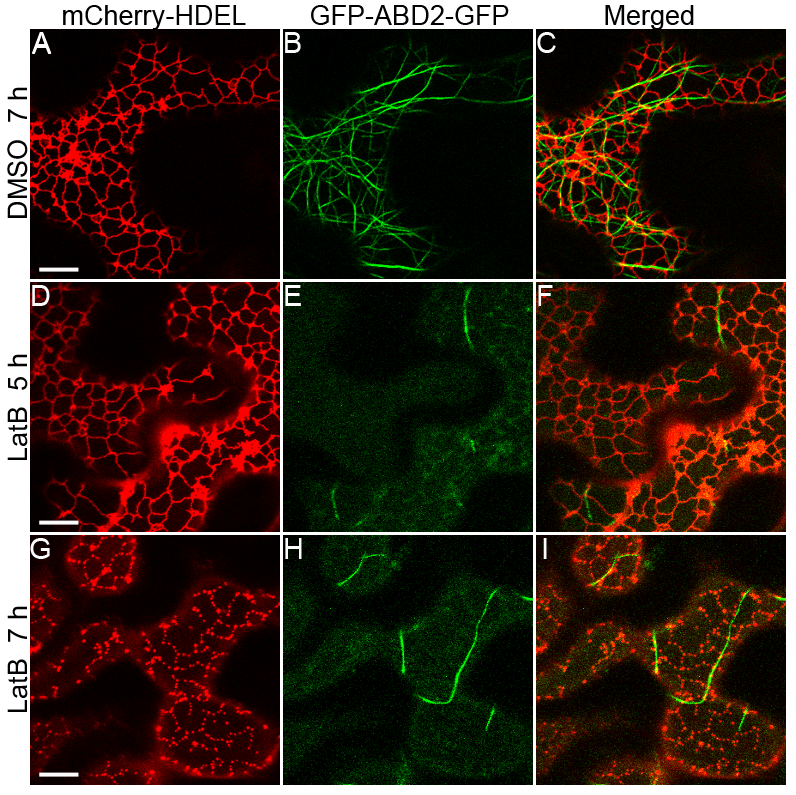

Supplement: S8 Fig — (A-C) The ER membrane and actin microfilament structure by DMSO control at 7 h post treatments. (D-I) The ER membrane and actin microfilament structure by 5 μM LatB at 5 h (D-F) or 7 h (G-I) post treatments. N. benthamiana was agroinfiltrated with the mCherry-HDEL and GFP-ABD2-GFP to label the ER network and actin microfilament, respectively. The cells were examined by confocal microscope. Bar, 10 μm. (TIF) [file ppat.1005443.s008.tif]

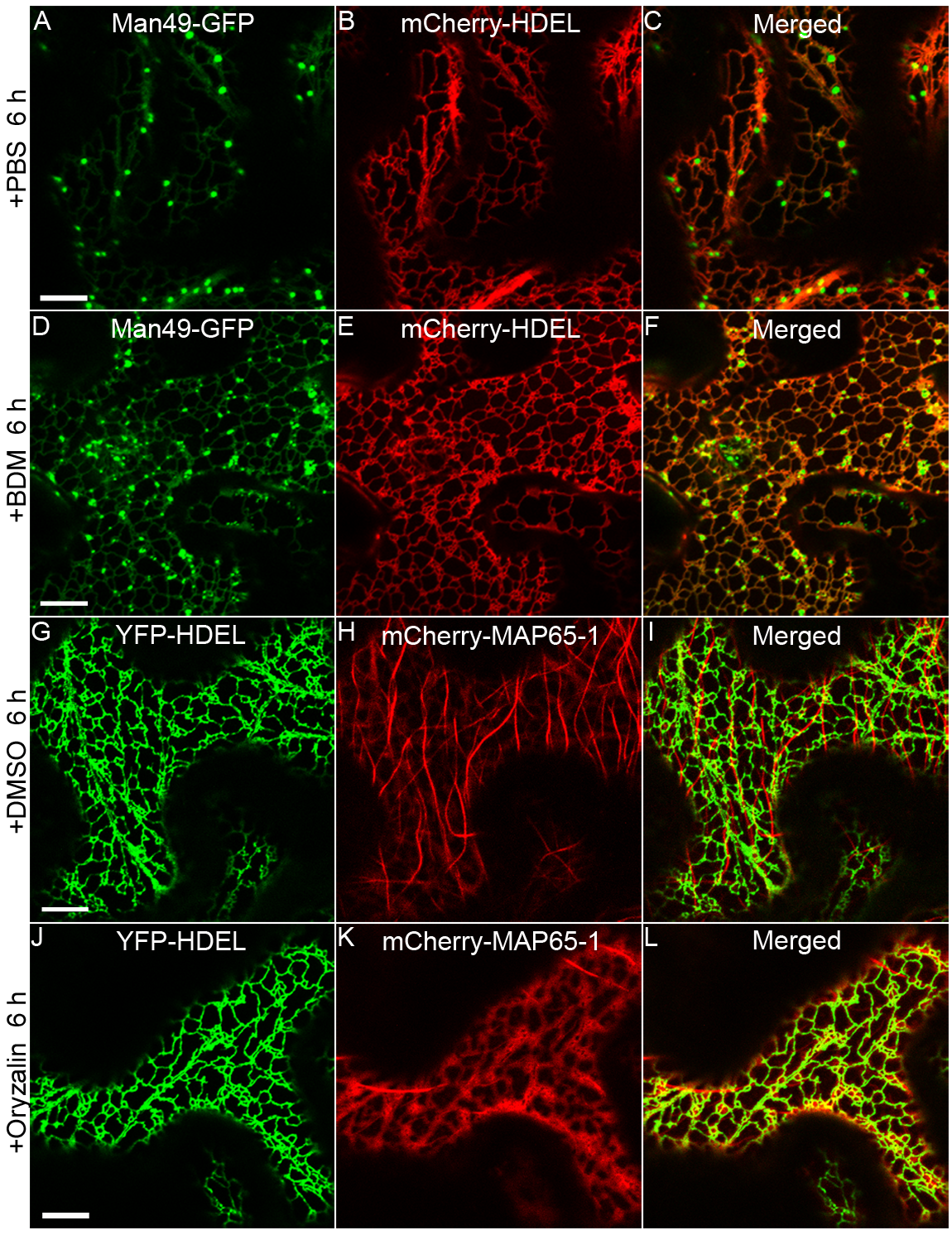

Supplement: S9 Fig — (A-F) The ER membrane and Golgi bodies structure by PBS control or by 100 mM BDM at 6 h post treatments. (G-L) The ER membrane and microtubule structure by DMSO control or by 20 μM oryzalin at 6 h post treatments. N. benthamiana was agroinfiltrated with the mCherry-HDEL/YFP-HDEL, Man49-mCherry and mCherry-MAP65-1, respectively, to label the ER network, Golgi bodies and microtubules. The cells were examined by confocal microscope. Bar, 10 μm. (TIF) [file ppat.1005443.s009.tif]

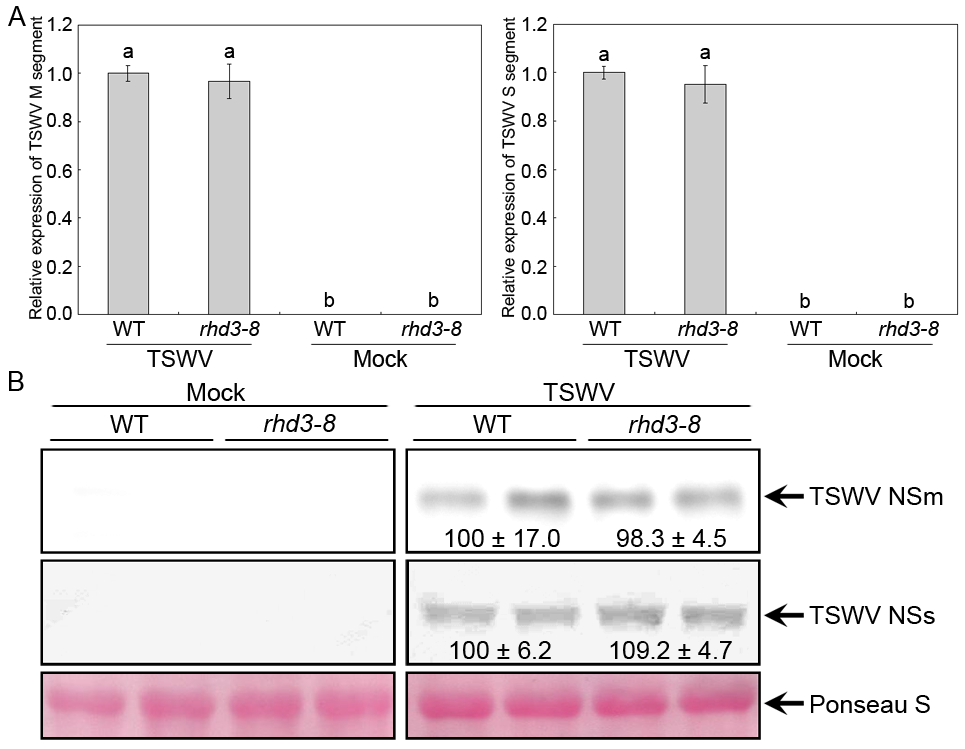

Supplement: S10 Fig — (A) Quantification of the accumulation of TSWV M (left panel) or the S (right panel) RNA segment in protoplasts of WT or rhd3 mutant by real-time RT-PCR. Primer pairs targeting NSm and NSs, respectively, were used to quantify the replication of the M and the S segment. (B) Expression level of TSWV nonstructural protein NSm (right upper panel) and NSs (right middle panel) in protoplasts of the WT or rhd3 mutant by immunoblotting. Protoplasts were isolated from fresh leaves of the WT or rhd3 mutant. Purified TSWV particles or PBS buffer (mock) were used to transfect protoplasts using PEG3350. Samples were collected 24 h after TSWV transfection for qRT-PCR or immunoblotting. (TIF) [file ppat.1005443.s010.tif]
