# Supplementary material for: The ER-Membrane Transport System Is Critical for Intercellular Trafficking of the NSm Movement Protein and Tomato Spotted Wilt Tospovirus
Source: PLoS Pathog. 2016 Feb 10;12(2):e1005443. doi: 10.1371/journal.ppat.1005443 (PMC4749231; doi:10.1371/journal.ppat.1005443)
Supplement: S1 Table — (DOC) [file ppat.1005443.s011.doc]

**S1 Table. Transmembrane (TM) or hydrophobic region (HR) analysis of TSWV NSm using different computational tools**

| **Algorithm** | **No. of TM or HRs (first aa/last aa)** |
| --- | --- |
| **MPEx** | 2 (127/145, 174/192) |
| **TMpred** | 1 (127/149) |
| **DAS** | 3 (130/139, 148/152, 174/182) |
| **TMHMM** | 0 TM; 2 HRs (127/158, 161/189) |
| **TopPred** | 1 (126/146) |
| **ΔG Prediction** | 0 TM; 2 HRs (127/153, 163/192) |
