# Supplementary material for: The ER-Membrane Transport System Is Critical for Intercellular Trafficking of the NSm Movement Protein and Tomato Spotted Wilt Tospovirus
Source: PLoS Pathog. 2016 Feb 10;12(2):e1005443. doi: 10.1371/journal.ppat.1005443 (PMC4749231; doi:10.1371/journal.ppat.1005443)
Supplement: S2 Table — (DOC) [file ppat.1005443.s012.doc]

**S2 Table. Time course analysis of cell-to-cell movement of NSm-GFP in leaf epidermis of *Nicotiana benthamiana* by bombardment**

| **Bombarded**  **plasmid** | **Hours post bombardment** | **Total signal**  **clusters** | **Number and percentage of total signal clusters** | |
| --- | --- | --- | --- | --- |
|  |  |  | **1 cell/cluster** | **≥2 cells/cluster** |
| **NSm-GFP** | 9~10 h | 49 | 41 (83.7%) ^a^ | 8 (16.3%) |
|  | 14~15 h | 55 | 36 (65.5%) | 19 (34.5%) |
|  | 19~21 h | 89 | 53 (59.6%) | 36 (40.4%) |
|  | 26~27 h | 86 | 50 (58.1%) | 36 (41.9%) |
|  | 46~48 h | 84 | 61 (72.6%) | 23 (27.4%) |
| **GFP-GFP** | 9~10 h | 54 | 54 (100%) | 0 |
|  | 14~15 h | 61 | 61 (100%) | 0 |
|  | 19~21 h | 68 | 68 (100%) | 0 |
|  | 26~27 h | 59 | 59 (100%) | 0 |
|  | 46~48 h | 52 | 52 (100%) | 0 |

^a^ Signal clusters comprise fluorescent cells, indicating presence of GFP fusion protein.
