# Supplementary material for: The ER-Membrane Transport System Is Critical for Intercellular Trafficking of the NSm Movement Protein and Tomato Spotted Wilt Tospovirus
Source: PLoS Pathog. 2016 Feb 10;12(2):e1005443. doi: 10.1371/journal.ppat.1005443 (PMC4749231; doi:10.1371/journal.ppat.1005443)
Supplement: S3 Table — (DOC) [file ppat.1005443.s013.doc]

**S3 Table. Cell-to-cell movement assay for GFP-GFP in leaf epidermis of *Nicotiana benthamiana* in the presence or the absence of NSm**

| **Bombarded plasmid** | **Agroinfiltration** | **Total foci** | **Number and percentage of total signal clusters** | |
| --- | --- | --- | --- | --- |
|  |  |  | **1 cell/cluster** | **≥2 cells/cluster** |
| **GFP-GFP** | Empty vector | 44 | 44 (100%) ^a^ | 0 |
|  | NSm | 33 | 33 (100%) | 0 |

^a^ Signal clusters comprise fluorescent cells, indicating presence of GFP fusion protein.
