# Supplementary material for: The ER-Membrane Transport System Is Critical for Intercellular Trafficking of the NSm Movement Protein and Tomato Spotted Wilt Tospovirus
Source: PLoS Pathog. 2016 Feb 10;12(2):e1005443. doi: 10.1371/journal.ppat.1005443 (PMC4749231; doi:10.1371/journal.ppat.1005443)
Supplement: S4 Table — (DOC) [file ppat.1005443.s014.doc]

**S4 Table. Cell-to-cell trafficking of NSm-GFP in *Nicotiana benthamiana* was not affected by interfering the ER-to-Golgi early secrection pathway or the cytoskeleton transport systems**

| **Bombarded plasmid** | **Treatment** | **Total foci** | **Number and percentage of total signal clusters** | | | | |
| --- | --- | --- | --- | --- | --- | --- | --- |
|  |  |  | **1 cell/cluster** | **2 cells/cluster** | **3 cells/cluster** | **≥4 cells/cluster** | ***P*-value ^b^** |
| **NSm-GFP** | DMSO | 60 | 39 (65.0%)^a^ | 7 (11.7%) | 2 (3.3%) | 12 (20.0%) |  |
|  | BFA ^c^ | 52 | 33 (63.5%) | 7 (13.4%) | 3 (5.8%) | 9 (17.3%) | >0.05 |
| **NSm-GFP** | PBS buffer | 54 | 38 (70.3%) | 1 (1.9%) | 5 (9.3%) | 10 (18.5%) |  |
|  | BDM ^d^ | 115 | 86 (74.8%) | 11 (9.6%) | 8 (7.0%) | 10 (8.7%) | >0.05 |
| **NSm-GFP** | DMSO | 78 | 60 (76.9%) | 5 (6.4%) | 4 (5.1%) | 9 (11.6%) |  |
|  | LatB ^e^ | 67 | 48 (71.6%) | 7 (10.4%) | 6 (9.0%) | 6 (9.0%) | >0.05 |
| **NSm-GFP** | DMSO | 72 | 52 (72.2%) | 5 (6.9%) | 4 (5.6%) | 11 (15.3%) |  |
|  | Oryzalin ^f^ | 87 | 63 (72.4%) | 6 (6.9%) | 7 (8.1%) | 11 (12.6%) | >0.05 |

^a^ Number and percentage (in parentheses) of fluorescent cells showing the presence of GFP fusion protein.

^b^ *P*-values were calculated using the unpaired two tailed Student *t*-test.

^c^ BFA was used at 2.5 μg/mL.

^d^ BDM was used at 100 mM.

^e^ LatB was used at 5 μM.

^f^ Oryzalin was used at 20 μM.
