# Supplementary material for: The ER-Membrane Transport System Is Critical for Intercellular Trafficking of the NSm Movement Protein and Tomato Spotted Wilt Tospovirus
Source: PLoS Pathog. 2016 Feb 10;12(2):e1005443. doi: 10.1371/journal.ppat.1005443 (PMC4749231; doi:10.1371/journal.ppat.1005443)
Supplement: S5 Table — (DOC) [file ppat.1005443.s015.doc]

**S5 Table. TSWV infection assay on wild-type (WT) and *rhd3-8* mutant plants of *Arabidopsis thaliana* from 7 to 27 days after inoculation (dpi)**

| Plants | **7** | **9** | **11** | **13** | **15** | **17** | **19** | **21** | **23** | **25** | **27** |
| --- | --- | --- | --- | --- | --- | --- | --- | --- | --- | --- | --- |
| **WT** | 0 a  (0%) ^b^ | 2  (15.4%) | 4  (30.8%) | 8  (61.6%) | 11  (84.6%) | 13  (100%) | 13  (100%) | 13  (100%) | 13  (100%) | 13  (100%) | 13  (100) |
| ***rhd3-8*** | 0  (0%) | 0  (0%) | 0  (0%) | 2  (15.4%) | 4  (30.8%) | 7  (53.8%) | 10  (76.9%) | 11  (84.6%) | 12  (92.3%) | 13  (100%) | 13  (100) |

^a^ Number of plants that developed disease symptoms.

^b^ Percentage of plants that developed disease symptoms among the total number of inoculated plants.
