# Supplementary material for: The ER-Membrane Transport System Is Critical for Intercellular Trafficking of the NSm Movement Protein and Tomato Spotted Wilt Tospovirus
Source: PLoS Pathog. 2016 Feb 10;12(2):e1005443. doi: 10.1371/journal.ppat.1005443 (PMC4749231; doi:10.1371/journal.ppat.1005443)
Supplement: S6 Table — (DOC) [file ppat.1005443.s016.doc]

**S6 Table. List of primers used in this study**

| **Clone** | **Primer** | | **Primer sequence (from 5' to 3')** | **Purpose** |
| --- | --- | --- | --- | --- |
| **p2300S-NSm** | F | XT654 | CGggatccGTATGTTGACTTTTTTTGGTA | To amplify TSWV NSm and clone into p2300S |
|  | R | XT655 | CCGctcgagCGGCCGCTATATCTCATCAAAAGATA |  |
| **p1300S-NSm-YFP** | F | XT664 | CGggatccATGTTGACTTTTTTTGGTA | To amplify TSWV NSm and clone into p1300S-YFP |
|  | R | XT665 | CGggatccTATCTCATCAAAAGATAACT |  |
| **pRTL2-TEV-NSm-GFP** | F | PV313 | GAGGACctcgagAATTCAACACAACATATACAAAAC | To amplify the TEV leader, NSm and GFP, respectively, then mix the three fragments and do overlap PCR to obtain TEV-NSm-GFP and clone into pRTL2 |
|  | R | PV326 | GGCTATCGTTCGTAAATGGTG |  |
|  | F | PV749 | CACCATTTACGAACGATAGCCATGTTGACTTTTTTTGGTA |  |
|  | R | P1092 | CCTCGCCCTTGCTCACCATggatccTATCTCATCAAAAGATAACT |  |
|  | F | XT379 | ATGGTGAGCAAGGGCGAGG |  |
|  | R | XT100 | GCGCGCgagctcTTACTTGTACAGCTCGTCC |  |
| **p1300S-TMV MP-3xHA**  **p1300S-TMV MP-mRFP** | F | XT771 | CGggatccATGGCTCTAGTTGTTAAAGG | To amplify TMV MP and clone into p1300S-3xHA or p1300S-mRFP |
|  | R | XT772 | CGggatccAAACGAATCCGATTCGGCGA |  |
|  | R | PV884’ | TGCAGCTGACAGCAGTTTTCTTGTTTTCTGCTGTCCCAAG |  |
|  | F | PV885’ | CTTGGGACAGCAGAAAACAAGAAAACTGCTGTCAGCTGCA |  |
|  | R | XT655 | CCGctcgagCGGCCGCTATATCTCATCAAAAGATA |  |
| **p2300S-NSm 133-135aa**  **IVI to DDD** | F | XT654 | CGggatccGTATGTTGACTTTTTTTGGTA | To amplify TSWV NSm fragments, then mix the two fragments and do overlap PCR to obtain the mutant and clone into p2300S |
|  | R | P1021 | CCTGGAAATCATCATGTATTGTTTTC |  |
|  | F | P1022 | GAAAACAATACATGATGATTTCCAGG*gatgacgat*TGGGTCTGCCCCACTATAC |  |
|  | R | XT655 | CCGctcgagCGGCCGCTATATCTCATCAAAAGATA |  |
| **p2300S-NSm**  **177-179aa**  **FVF to DDD** | F | XT654 | CGggatccGTATGTTGACTTTTTTTGGTA | To amplify TSWV NSm fragments, then mix the two fragments and do overlap PCR to obtain the mutant and clone into p2300S |
|  | R | P1025 | ACAGATAGGATCAGTTATTGTCC |  |
|  | F | P1026 | GGACAATAACTGATCCTATCTGT*gatgacgat*TATCTGAACTGGTCTATTCCG |  |
|  | R | XT655 | CCGctcgagCGGCCGCTATATCTCATCAAAAGATA |  |
| **pRTL2-TEV-NSm-GFP 133-135aa**  **IVI to DDD** | F | PV313 | GAGGACctcgagAATTCAACACAACATATACAAAAC | To amplify the TEV, NSm mutant and GFP, respectively, then mix the three fragments and do overlap PCR to obtain TEV-NSm-GFP and clone into pRTL2 |
|  | R | PV326 | GGCTATCGTTCGTAAATGGTG |  |
|  | F | PV749 | CACCATTTACGAACGATAGCCATGTTGACTTTTTTTGGTA |  |
|  | R | P1021 | CCTGGAAATCATCATGTATTGTTTTC |  |
|  | F | P1022 | GAAAACAATACATGATGATTTCCAGG*gatgacgat*TGGGTCTGCCCCACTATAC |  |
|  | R | P1092 | CCTCGCCCTTGCTCACCATggatccTATCTCATCAAAAGATAACT |  |
|  | F | XT379 | ATGGTGAGCAAGGGCGAGG |  |
|  | R | XT100 | GCGCGCgagctcTTACTTGTACAGCTCGTCC |  |
| **pRTL2-TEV-NSm-GFP 177-179aa**  **FVF to DDD** | F | PV313 | GAGGACctcgagAATTCAACACAACATATACAAAAC | To amplify the TEV, NSm mutant and GFP, respectively, then mix the three fragments and amplify with overlap PCR to obtain TEV-NSm-GFP and clone into pRTL2 |
|  | R | PV326 | GGCTATCGTTCGTAAATGGTG |  |
|  | F | PV749 | CACCATTTACGAACGATAGCCATGTTGACTTTTTTTGGTA |  |
|  | R | P1025 | ACAGATAGGATCAGTTATTGTCC |  |
|  | F | P1026 | GGACAATAACTGATCCTATCTGT*gatgacgat*TATCTGAACTGGTCTATTCCG |  |
|  | R | P1092 | CCTCGCCCTTGCTCACCATggatccTATCTCATCAAAAGATAACT |  |
|  | F | XT379 | ATGGTGAGCAAGGGCGAGG |  |
|  | R | XT100 | GCGCGCgagctcTTACTTGTACAGCTCGTCC |  |
|  | R | PV884 | TGCAGCTGACAGCAGTTTTCTTGTTTTCTGCTGTCCCAAG |  |
|  | F | PV885 | CTTGGGACAGCAGAAAACAAGAAAACTGCTGTCAGCTGCA |  |
|  | R | XT665 | CGggatccTATCTCATCAAAAGATAACT |  |
| **p1300S-NSm^F4A/F5A^-YFP** | F | PV626 | CGGGATCCATGTTGACTGC*t*GC*t*GGTAATAAGGGGTCTTCTAAG | To amplify TSWV NSm mutant and clone into p2300S-YFP |
|  | R | XT655 | CCGctcgagCGGCCGCTATATCTCATCAAAAGATA |  |
| **p1300S-**  **NSm^D230A/D232A^-YFP** | F | XT664 | CGggatccATGTTGACTTTTTTTGGTA | To amplify TSWV NSm fragments, then mix the two fragments and do overlap PCR to obtain the mutant and clone into p2300S-YFP |
|  | R | PV624 | CAGCTCTGGGTGAATCACAA |  |
|  | F | PV625 | TTGTGATTCACCCAGAGCTG*c*TAAAG*c*CAAAAGTTGCATGGTCATACC |  |
|  | R | XT665 | CGggatccTATCTCATCAAAAGATAACT |  |
| **pRTL2-TEV-**  **NSm^F4A/F5A^-GFP** | F | PV313 | GAGGACctcgagAATTCAACACAACATATACAAAAC | To amplify the TEV, NSm mutant and GFP, respectively, then mix the three fragments and do overlap PCR to obtain TEV-NSm-GFP and clone into pRTL2 |
|  | R | PV326 | GGCTATCGTTCGTAAATGGTG |  |
|  | F | PV749’ | CACCATTTACGAACGATAGCCATGTTGACTGC*t*GC*t*GGTAATAAGGGGTCTTCTAAG |  |
|  | R | P1092 | CCTCGCCCTTGCTCACCATggatccTATCTCATCAAAAGATAACT |  |
|  | F | XT379 | ATGGTGAGCAAGGGCGAGG |  |
|  | R | XT100 | GCGCGCgagctcTTACTTGTACAGCTCGTCC |  |
| **pRTL2-TEV-**  **NSm^D230A/D232A^-GFP** | F | PV313 | GAGGACctcgagAATTCAACACAACATATACAAAAC | To amplify the TEV, NSm mutant and GFP, respectively, then mix the three fragments and amplify with overlap PCR to obtain TEV-NSm-GFP and clone into pRTL2 |
|  | R | PV326 | GGCTATCGTTCGTAAATGGTG |  |
|  | F | PV749 | CACCATTTACGAACGATAGCCATGTTGACTTTTTTTGGTA |  |
|  | R | P1092 | CCTCGCCCTTGCTCACCATggatccTATCTCATCAAAAGATAACT |  |
|  | F | XT379 | ATGGTGAGCAAGGGCGAGG |  |
|  | R | XT100 | GCGCGCgagctcTTACTTGTACAGCTCGTCC |  |
|  | R | PV624 | CAGCTCTGGGTGAATCACAA |  |
|  | F | PV625 | TTGTGATTCACCCAGAGCTG*c*TAAAG*c*CAAAAGTTGCATGGTCATACC |  |
| **pRTL2-GFP** | F | XT1007 | GGccatggTGAGCAAGGGCGAGG | To amplify the GFP and clone into pRTL2 |
|  | R | XT1008 | CGtctagaTTACTTGTACAGCTCGTCC |  |
| **pRTL2-GFP-GFP** | F | PV313 | GAGGACctcgagAATTCAACACAACATATACAAAAC | To amplify the GFP and clone into pRTL2-GFP |
|  | R | P1686 | CATGccatggCCTTGTACAGCTCGTCCATG |  |
| **mCherry-HDEL // NSm-GFP** | NSm-GFP cassette was cut from pRTL2-TEV-NSm-GFP, inserted into ER-rb *CD3-960* by HindIII. | | | |
| **mCherry-HDEL // GFP-GFP** | GFP-GFP cassette was cut from pRTL2-GFP-GFP, inserted into ER-rb *CD3-960* by HindIII. | | | |
| **Real-time PCR analysis** | F | P1675 | TCGGCAACGGGAAGCAAAA | To quantify the expression of TSWV M segment |
|  | R | P1676 | GGGGCAGACCCATATCACAAT |  |
| **Real-time PCR analysis** | F | P1677 | ACTCTGTTCTGGCACTATCTGTTTG | To quantify the expression of TSWV S segment |
|  | R | P1678 | GGAGCTGGAATCGGTCTGTAATA |  |
| **Real-time PCR analysis** | F | P1305 | GCTGACCGTATGAGCAAAGA | To amplify *Arabidopsis* actin2, used as internal control |
|  | R | P1306 | ATCTGCTGGAATGTGCTGAG |  |
| **NSm HR1** | F | F1 | CTAGTGGAGGTCCTGGATACATGATGATCTCCCGCATCGTCATTTGGGTGTGCCCCACCATCCCCAAC | *in vitro* translation and membrane insertion assay.  a codon-optimized HR1 based on reticulocyte codon usage. |
|  | F | F2 | CCCACCGGCAAGCTGGTGGTCGCCCTGGTGGGACCTGGAGGGGTAC |  |
|  | R | R1 | CACCCAAATGACGATGCGGGAGATCATCATGTATCCAGGACCTCCA |  |
|  | R | R2 | CCCTCCAGGTCCCACCAGGGCGACCACCAGCTTGCCGGTGGGGTTGGGGATGGTGGGGCA |  |
| **NSm HR2** | F | F3 | CTAGTGGAGGTCCTGGAATCATTCTGAAGGGGCAGGGCACCATCACCGACCCCATCTGC | *in vitro* translation and membrane insertion assay.  a codon-optimized HR2 based on reticulocyte codon usage. |
|  | F | F4 | TTCGTGTTCTACCTGAACTGGTCCATTGGACCTGGAGGGGTAC |  |
|  | R | R3 | GGTGCCCTGCCCCTTCAGAATGATTCCAGGACCTCCA |  |
|  | R | R4 | CCCTCCAGGTCCAATGGACCAGTTCAGGTAGAACACGAAGCAGATGGGGTCGGTGAT |  |

Lowercase letters are the restriction enzyme sites, NSm sequence is underlined, and mutated sequence of NSm is the lowercase italic letters.
